# A Minimalist Model Lipid System Mimicking the Biophysical Properties of Escherichia coli’s Inner Membrane

**DOI:** 10.1021/acs.langmuir.5c01138

**Published:** 2025-05-07

**Authors:** Nicolo Tormena, Teuta Pilizota, Kislon Voïtchovsky

**Affiliations:** † Physics Department, 3057 Durham University, South Road, Durham DH1 3LE, U.K.; ‡ School of Biological Sciences and Centre for Engineering Biology, 2152 The University of Edinburgh, Alexander Crum Brown Road, Edinburgh EH9 3FF, U.K.; § Department of Physics, University of Cambridge, JJ Thompson Avenue, Cambridge CB3 0HE, U.K.

## Abstract

Biological membranes are essential for the development and survival of organisms. They can be highly complex, usually comprising a variety of lipids, proteins, and other biomolecules organized around a lipid bilayer structure. This complexity makes studying specific features of biological membranes difficult, with many research studies relying on simplified models, such as artificial vesicles or supported lipid bilayers. Here, we search for a minimal, lipid-only model system of the Escherichia coli inner membrane. We aim to retain the main lipidomic components in their native ratio while mimicking the membrane’s thermal and mechanical properties. Based on previous studies, we identify 18 potential model systems reflecting key aspects of the known lipidomic composition and progressively narrow down our selection based on the systems’ phase transition temperature and mechanical properties. We identify three ternary model systems able to form stable bilayers that can be made of the commercially available synthetic lipids 16:0–18:1 phosphatidylethanolamine (POPE), 16:0–18:1 phosphatidylglycerol (POPG), and 16:0–18:1 cardiolipin (CL). We anticipate our results to be of interest for future studies making use of E. coli models, for example, investigating membrane proteins’ function or macromolecule–membrane interactions.

## Graphical abstract

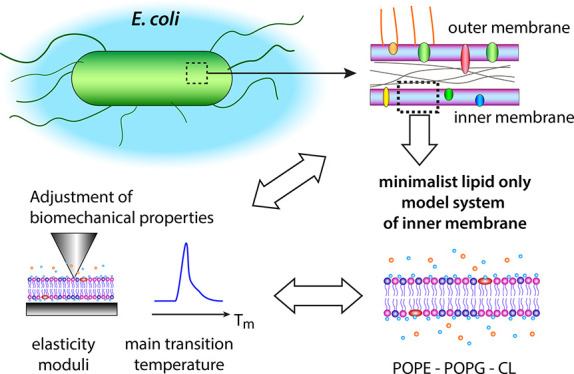

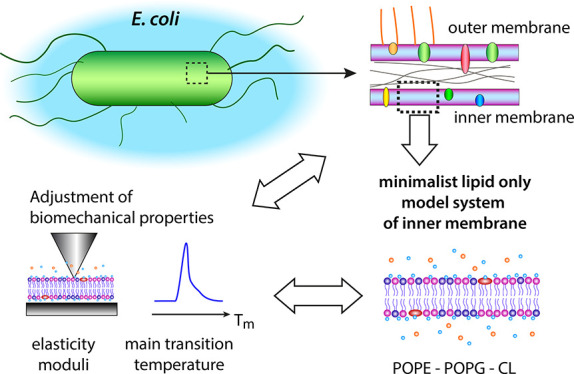

## Introduction

All types of organisms, from prokaryotic to eukaryotic, separate their internal environment from the exterior using biological membranes that consist of a self-assembled and self-synthesized double layer of phospholipids with a hydrophobic matrix in which a large number of proteins and sugars are bound or embedded.^1^ The function of biological membranes is multifold, first acting as a physical barrier, but also serving as a unique environment for many biological processes.^2–5^

The prokaryotic cell envelope consists not only of the cell membrane(s) but also of a cell wall, a structural layer made of a peptidoglycan matrix.^6^ In Gram-positive bacteria, the cell envelope is composed of one plasma membrane and a thick external peptidoglycan layer, while Gram-negative bacteria are characterized by a thinner cell wall that separates two phospholipid membranes, called inner and outer membranes, respectively.^7^ Because of its fast growth rate, genetic simplicity, and ease of culturing, Gram-negative Escherichia coli is one of the most common and well-studied model organisms and, as such, is of great importance for biotechnology and human health.^8–14^ E. coli’s inner and outer membranes present a plethora of specific proteins, and the inner membrane acts as a capacitor for ions, allowing the generation of electrochemical gradients that contribute to powering the cell.^2^ Both membranes share a broadly similar phospholipid composition, with slight variations in phosphate headgroup and acyl chain distribution.^15–20^ The outer membrane also contains lipopolysaccharides (LPS), which are absent in the inner membrane and contribute to its defensive and permeability functions. The composition and biophysical properties of the membranes are known to adapt to the environment^15–17^ and have been demonstrated to have compositional asymmetry between the two lipid leaflets.^21,22^ These features add to the challenge of developing a suitable model E. coli membrane system, with various studies often taking different approaches.^23–32^ For example, some studies omit cardiolipin, a key component of E. coli membrane,^23,24^ while others use a wide range of phospholipid headgroups with different alkyl chain lengths,^25–30^ with or without cardiolipin. Furthermore, the biophysical properties of the model membranes are rarely investigated despite being crucial for the structural stabilization, active functionality, and localization of membrane proteins.^31,32^ Given the complexity of E. coli membranes and their dependence on the environment, even the best model systems are unlikely to capture all of the native membrane’s properties, but broadly replicating the native lipidomic ratios and biophysical properties would already provide a valuable basis for a wide range of studies.^33,34^

This is the aim of the present study. Our strategy relies on previous lipidomic studies^15–20,35,36^ to identify binary and ternary lipid combinations that recapitulate the main aspects of E. coli’s inner membrane lipidic composition and stoichiometry at standard growth temperature (37 °C). We then narrow down possible models by comparatively testing their thermal and mechanical properties against the native membrane using a combination of differential scanning calorimetry (DSC), atomic force microscopy (AFM), and optical microscopy. The main transition temperature (*T*_m_) is used to quantify the system’s thermal characteristics, noting that *T*_m_ also offers good indications of how the membrane behaves mechanically: at *T*_m_, the thermal energy overcomes the internal energy of the membrane, which is dominated by the average interlipid interaction energy. The internal energy influences both molecular order and dynamics within the membrane,^37^ and hence the propagation of any imposed mechanical stress. We directly access the mechanical properties of the membranes by measuring the elastic modulus *Y*, and from it, we estimate the stretching modulus *K*_a_, equivalent to the first Lamé coefficient. This is justified by the fact that the Helmholtz free energy of the E. coli’s inner membrane consists of the stretching and turgor pressure energies,^38^ with the bending energy being generally negligible^38^ and the fact that fluid membranes cannot support in-plane shear.^39^ Practically, we narrow down 18 possible combinations of the three main lipids present in E. coli and identify three mixtures that form stable and reproducible E. coli model membrane systems with the desired biophysical characteristics.

## Materials and Methods

All chemicals and lipids were obtained from commercial sources and used without further purification.

### Lipids

All of the lipids were purchased from Avanti Polar Lipids (Alabaster, AL). The following lipids were purchased and dissolved in chloroform: 1-palmitoyl-2-oleoyl-*sn*-glycero-3-phosphoethanolamine (POPE), 1-palmitoyl-2-oleoyl-*sn*-glycero-3-phospho-(1’-rac-glycerol) (POPG), and 1’,3′-bis­[1-palmitoyl-2-oleoyl-*sn*-glycero-3-phospho]-glycerol (CL). 1,2-Dipalmitoyl-*sn*-glycero-3-phospho-(1’-rac-glycerol) (DPPG) was obtained in powder form. The native E. coli membranes (37 °C growth) were obtained as E. coli Extract Polar (comprising only the polar lipids component) and E. coli Total Extract (full lipid extract) already dissolved in a chloroform–methanol solution. These membranes were obtained from E. coli B (ATCC 11303) grown in Kornberg Minimal media at 37 °C, as described by Avanti. We note that most studies on E. coli membrane lipidomic composition, as well as the majority of modern-day microbiology studies, rely on K-12 strain isolates. These two commonly used E. coli strains present highly similar genomes, which mainly diverge in their proteomic profiles,^40,41^ but no alterations have been detected in their lipidomic profile nor in the key proteins involved in phospholipid synthesis, confirming the highly conserved composition of E. coli membrane within different strains.

### Chemicals

Salts (all >99% purity) were purchased from Sigma-Aldrich (Dorset, UK) and dissolved/diluted in ultrapure water (at 18 MΩcm obtained from Merck-Millipore, Watford, UK). A MOPS buffer-based solution was prepared with specific ion concentrations as follows: 50 mM NaCl, 9.5 mM NH_4_Cl, 0.5 mM MgCl_2_, 0.3 mM K_2_SO_4_, and 1 μM CaCl_2_–2H_2_O. The pH was adjusted to 6.5 prior to mixing with lipids.

### Large Multilamellar Vesicles Preparation

Lipids dissolved in chloroform were mixed following the appropriate molar ratios into a 4 mL glass vial, predried under a gentle nitrogen flow, and fully dried overnight in a vacuum chamber. Large multilamellar vesicles were obtained by freeze-thawing.^42,43^ Briefly, the lipid film was rehydrated in 2 mL of a MOPS buffer-based solution to obtain a lipid concentration of 10 mg/mL and then briefly heated while sonicating in a sonication bath. Subsequently, the vial was frozen (left in the freezer for 15 min). This heating-freezing process was repeated for 6 consecutive cycles to successfully form large multilamellar vesicles (LMVs), which was confirmed by the lower turbidity of the solution and optical microscopy imaging.

### Unilamellar Vesicles Preparation

Lipids were mixed and dried following the same protocol as that used for LMV preparation. The lipid film was subsequently rehydrated in 1 mL of MOPS buffer-based solution, obtaining a lipid concentration of 1 mg/mL. The vial was gently bath-sonicated for 15 min at a temperature 5–10 °C higher than the highest *T*_m_ of the lipid species in the mixture until the solution appeared milky, indicating the formation of multilamellar vesicles. For small unilamellar vesicles (SUVs), the solution was extruded 31 times using a Mini-Extruder kit (Avanti Polar Lipids) with a Whatman 100 nm nucleopore membrane (GE Healthcare Life Sciences, Little Chalfont, UK).

### Supported Lipid Bilayers Preparation

The SUV solution was diluted 5 times to reach a 0.2 mg/mL concentration. 100 μL of the SUV solution was deposited on a disk of grade 1 freshly cleaved Muscovite mica (SPI Supplies, West Chester, PA, USA). The disk, already mounted on the AFM stage, was left to incubate for 20 min at 50 °C, covered with a Petri dish. The sample was then gently rinsed with a MOPS buffer to remove any unfused lipid SUVs. The temperature was then cooled to 40 °C and equilibrated for 15 min, as a starting point for the measurement. The salt concentration in the MOPS buffer was sufficient to ensure the formation of a spread and uniform supported lipid bilayer (SLB) system over the flat mica surface.

### Differential Scanning Calorimetry (DSC)

To observe the lipids’ main melting transition and extract the associated melting temperature values, DSC measurements were performed on a DSC 2500 (TA Instruments, Delaware, USA). Preliminary DSC heating tests were performed to identify ideal lipid concentration and DSC scan parameters, and to ensure a satisfactory signal-to-noise ratio and reproducibility of the data (Figure S1). With DSC, faster scan rates tend to provide a better signal-to-noise ratio but may result in potentially lower temperature resolution. DSC test runs on binary lipid mixtures were performed with increasing lipid concentrations (from 1 mg/mL up to 10 mg/mL) and heating scan rates (from 2 °C/min up to 10 °C/min) while maintaining the temperature scanning range of −10 °C to 60 °C (Figure S1A,B). Since the scan rate can shift the experimental melting point, DSC cooling experiments were performed with the same increasing scan rates (from 2 °C/min up to 10 °C/min) and temperature range (Figure S1C,D). This enables us to infer the melting point of our reference mixture at a 0 °C/min scan rate (thermodynamic equilibrium) and therefore quantify the effect of the scan rate on the experimental melting point (Figure S1E). The dependence of the measured transition temperature on the scan rate is not trivial, with previous studies^44,45^ reporting the following nonlinear behavior:

1 $$T_{m,\beta }=T_{m}+B\beta^{z}$$

where *T*_m,β_ is the measured melting temperature at each scan rate, *T*_m_ is the equilibrium or “true” melting temperature, β is the scan rate, and *B* and *z* are fitting parameters. Experiments run at different scan rates enabled us to determine *B* and *z* to subsequently correct all the measured transition temperatures (see fits in Figure S1E).

All other experiments were performed as follows: 10 μL of LMV solutions at 10 mg/mL were loaded into the calorimeter, and a heating rate of 5 °C/min was used in a temperature range of −10 °C to 60 °C. Samples were equilibrated for 5 min at the starting temperature (−10 °C) before starting the measurement. Three independent samples of each mixture were analyzed separately to ensure reliable statistical analysis.

### Optical Brightfield Microscopy

Images of LMVs were taken using an Eclipse E200 (Nikon) microscope with 10× and 40× phase-contrast objectives. Vesicles were imaged in a tunnel slide prepared as previously reported.^3,46^ Briefly, two parallel strips of double-sided sticky tape were positioned onto a microscope slide and covered with a 22 × 40 mm cover glass, which was then pressed against the tape to form a tunnel. Approximately 10 μL of vesicles in solution was added to the tunnel for imaging. Pixel size was calibrated using a coverslip presenting a 10 mm × 10 mm square grid with 0.1 mm spacing (Graticules Optics).

### Atomic Force Microscopy

Imaging was conducted using a commercial Cypher ES AFM (Oxford Instruments, Santa Barbara, CA, USA), equipped with temperature control. SNL-10 cantilevers (Bruker Scientific Instruments, Billerica, MA, USA) with a nominal spring constant of 0.35 N/m were used. The tip has a pyramidal shape with a tip radius of less than 12 nm at its apex. AFM imaging was performed in amplitude modulation, fully immersing the sample and cantilever/tip in liquid. The cantilever was acoustically oscillated at a frequency close to its resonance in liquid (∼10 kHz), with images acquired while the oscillating tip raster-scanned the surface while keeping the oscillation amplitude constant.

Force spectroscopy mapping was conducted in contact mode (no acoustic excitation). A schematic illustration of the measurement principle is shown in Figure S2. A force map was created from 1024 force curves (32 × 32) over a 25 μm^2^ area. Calibration of the cantilever’s spring constant was performed by first determining its inverse optical lever sensitivity from a force–distance curve acquired on a stiff surface (bare mica). The spring constant of each cantilever was subsequently determined from its thermal spectrum.^47^ This allowed for accurate derivation of the Young's modulus*Y* and of membrane rupture force *F*_r_. For the analysis, we used the following relationship between the force *F*_sphere_ applied by the tip (assumed locally spherical), the indentation depth δ of the tip into the membrane, *R* the radius of curvature of the tip, and the membrane thickness *h*:^48^

2 $$F_{\mathrm{sphere}}=\frac{16}{9}Y\sqrt{{R\delta }^{3}}\left[1+\frac{1.133\sqrt{\delta R}}{h}+\frac{1.497\delta R}{h^{2}}+\frac{1.469\delta R\sqrt{\delta R}}{h^{3}}+\frac{0.755\left(\delta^{2}R^{2}\right)}{h^{4}}\right]$$

where δ ≤ *R*. This formula was derived assuming a thin membrane on a much stiffer substrate and that the Poisson ratio, which couples in-plane and out-of-plane strain, is exactly *v* = 0.5. In other words, the membrane is assumed incompressible with its volume conserved under compression. This assumption is common for biosystems^49^ and usually a good approximation, but it is not necessarily exact.^50,51^ In practice, the indentation of the membrane is carried out with an AFM tip, ensuring a linear indentation regime^52^ to derive *Y*. Both *Y* and *F*_r_ were obtained using the same tip for all the measurements to ensure direct comparability between the results, regardless of any possible systematic offset. The emphasis is hence not placed on the absolute stiffness values^53,54^ but rather on the relative differences between samples, with the two phases yielding the expected bimodal distribution. All of the force maps over all the different samples were acquired with the same AFM cantilever/tip. The cantilever was cleaned with isopropanol and ultrapure water and calibrated before a new measurement was started to check its conditions. Moreover, to ensure the reliability of the results, AFM images were taken before and after the measurements, comparing the membrane’s topographical features.

### Data Analysis

DSC results were analyzed with the TRIOS software provided by the instrument’s manufacturer. The software was used to correct the thermogram baselines and then obtain *T*_m_ at the highest point of the calorimetric peak. DLS data were analyzed using the Zetasizer Family software v.8.01, provided by the instrument’s manufacturer. The size of the vesicles and the associated uncertainty (standard deviation) were obtained by fitting the experimental size histogram with a Gaussian distribution. AFM images and topographical information (section profiles) were obtained using Gwyddion,^55^ an open-source modular software for scanning probe microscopy data visualization and analysis. Optical microscopy images were analyzed using the open-source image processing package ImageJ/FIJI.^56^ Force spectroscopy data from indentation measurements were analyzed using bespoke routines programmed in Igor Pro (WaveMetrics, Lake Oswego, OR, USA) and Python.^57^

## Results and Discussion

### PE, PG, and CL Are the Main Lipid Species in E. coli’s Membrane

The composition of E. coli’s inner membrane obtained from previous lipidomic and mass spectroscopy assays is summarized in Table 1. Where available, melting temperatures obtained from previous calorimetric studies^15,17,58^ are also shown. To control the thermal and mechanical properties of the model system, we need to consider: (i) the lipid polar head distribution, which controls lipid–lipid interactions and charge distribution along the surface; (ii) the acyl chain length, which controls membrane thickness, fluidity, and membrane packing; and (iii) the acyl chain saturation degree, since it regulates lipid packing within the bilayer. *T*_m_ values are typically 7–16 °C lower than the growth temperature in a specific medium because E. coli membranes are fluid, dynamic, and able to rearrange their composition through epigenetic reprogramming.

**Table 1:** Summary of the E. coli Inner Membrane’s Composition and Properties, When Grown at the Standard Growth Temperature of 37 °C (Upper Part) and Variation of E. coli Membrane Melting Temperature Based on Growth Temperature (Lower Part)^a^

Polar head distribution		Lipid chain length	
Lipid species	Concentration (%)	Chain length	Concentration (%)
PE	75 ^16,18^ 70–78,^35^ ∼ 80,^36^ 81.7,^20^ 62 ^59^	12C	0 ^16^
PG	19,^16^ 20,^18^ 11–18,^35^ ∼ 15,^36^ 6.5,^20^ 14 ^59^	14C	4,^16^ 1–3,^35^ 3–5,^58^ 4.7 ^20^
CL	7–12,^35^ ∼ 5,^36^ 24 ^59^	16C	74.7,^16^ 63–73,^35^ 43–74,^58^ 67.1 ^20^
		17C	7–22,^35^ 7–29,^58^ 4.4 ^20^
		18C	16.3,^16^ 8–21,^35^ 19,^58^ 23.3 ^20^

Chain saturation degree		E. coli membrane melting temperature	
Saturation degree	Concentration (%)	Growth *T* (°C)	*T*_m_ (°C)
Saturated	48.5,^16^ 46–38.9,^60^ 47–56,^35^ 39.4 ^20^	17	10,^15^ 10 ^17^
Unsaturated	46.9,^16^ 32.4–3.7,^60^ 44–53,^35^ 64–52,^58^ 60.6 ^20^	27	15 ^15^
Cyclized	0.7–32.5 ^60^	37	28.5,^15^ 21.3,^17^ 25<^58^

a The data compile results obtained from published literature.

Here, we focus on mimicking the inner membrane of E. coli grown at physiological temperature (37 °C). The *T*_m_ of the model membrane should therefore be lower than 30 °C while maintaining the lipid polar headgroup ratios, chain length, and overall degree of saturation as close as possible to that of the native membrane. From Table 1, the reported compositional ratios of E. coli inner membrane vary by up to 20%,^16,18,35,36,59^ but all studies suggest that the main lipid species are phosphatidylethanolamines (PEs, 60–80% molar ratio) followed by phosphatidylglycerols (PGs, 15–30% molar ratio) and other minor lipids. Within these minor species, the most abundant is cardiolipin (CL, ∼5%), which, due to its unusual structure (see Figure S3), plays a crucial role in the membrane’s physiological behavior, including mechanotransduction.^61–63^ We, therefore, include CL in our candidate model membranes. Apart from CL, most lipids in E. coli’s inner membrane show long carbon chains (>16C), with an even distribution between saturated and unsaturated lipid chains. Cyclized lipid chains are also found in relatively high concentrations in the inner membrane^60^ and are known to significantly reduce the lipid packing density.^25^ Mass spectroscopy results show that the three most common lipid chains are C16:0, C18:1, and the cyclized C17:1 (cyC17:1).^64^ The cyclized lipids have been reported to influence membrane fluidity thanks to their cyclic motifs.^65^ However, because cyclized lipids are rare in other organisms and not commercially available, and in keeping with our goal of a simple model system, we focus here on more common noncyclized lipids. This limitation is mitigated by the inclusion of CL, which has been shown to exert similar fluidity modulation^66,67^ as other bacterial-specific lipids such as hopanoids.^68^ Moreover, CL introduces additional biologically relevant features, including membrane curvature regulation^69^ and the formation of protein-interaction motifs,^62^ representing a crucial element for the E. coli membrane function.

Based on our literature review, we devised 18 different lipid mixtures by systematically varying the stoichiometries of the four main lipid types in 5% steps around the reported values for E. coli’s inner membrane grown at 37 °C (Table 2). For simplicity, we used binary and ternary mixtures of POPE, POPG (both lipids with 16:0–18:1 chains), DPPG (16:0 chains), and CL (2 chains 16:0 and 2 chains 18:1), which all theoretically match the structural requirements for native E. coli inner membrane. POPE and CL were selected for their acyl chain compositions, which closely match the natural pattern of the E. coli inner membrane (Table 1), and represent two of the most common lipid chains in this bilayer.^64^ On the other hand, both POPG and DPPG could serve as the PG source for the E. coli-like model because they exhibit the most common acyl chains in these bacteria. POPG is often used for its relatively low *T*_m_ (−2 °C), thus preventing phase separation in the membrane or the formation of ordered, raft-like domains. In contrast, DPPG has a transition temperature of *T*_m_ = 41 °C, which is more likely to induce phase separation, but its two saturated acyl chains bring the overall molar ratio of unsaturated chains closer to the native ratio. Since the charged headgroup that characterizes the PG family can, in principle, help prevent phase separation of DPPG, we kept both POPG and DPPG in our candidate model system.

**Table 2:** Candidate Lipid Mixtures to Be Used as Model Systems for E. coli’s Inner Membrane, When Grown at Physiological Temperature (37 °C)^a^

Mixture name	POPE (%)	POPG (%)	DPPG (%)	CL (%)
1-A	80	20	0	0
1-B	80	0	20	0
2-A	75	25	0	0
2-B	75	0	25	0
3-A	70	30	0	0
3-B	70	0	30	0
4-A	60	40	0	0
4-B	60	0	40	0
5-A	80	15	0	5
5-B	80	0	15	5
6-A	75	20	0	5
6-B	75	0	20	5
7-A	70	25	0	5
7-B	70	0	25	5
8-A	70	20	0	10
8-B	70	0	20	10
9-A	65	25	0	10
9-B	65	0	25	10

a Each mixture contains two or three types of phospholipids: POPE, POPG, DPPG, and CL (16:0–18:1). Mixtures are indicated with a number and a letter, with the number indicating a specific lipid molar ratio and the letter indicating the specific PG lipid used (“A” being POPG and “B” being DPPG).

### POPG Ternary Mixtures Successfully Mimic E. coli Inner Membrane Transition Temperature

Having identified candidate model membranes in Table 2, we next determined their melting temperatures. We used lipidic LMV samples in a MOPS buffer-based solution (see Materials and Methods) and extracted *T*_m_ for each sample from the DSC transition peak. The composition of MOPS matches the salt concentrations of commonly used E. coli growth media^70–72^ but lacks the nutrients. LMVs are routinely used for this type of measurement because they enhance the signal-to-noise ratio (SNR) compared to other types of lipid vesicles.^42,73–77^ We selected a heating rate of 5 °C/min for optimal SNR and corrected it for kinetic effects^44,45^ to infer the “true” (i.e., thermodynamic equilibrium) *T*_m_ value (see Materials and Methods and Figure S1 for more details). We also analyzed two commercial E. coli membrane extracts that we refer to as E. coli “Native” and E. coli “Polar Extract”. E. coli Native is a direct lipid extract from both E. coli membranes, while E. coli Polar Extract has been further purified to remove unknown lipid species. The PE and PG ratios in Polar align closely with most literature-reported values for the inner membrane. However, a recent study on the PE composition of E. coli reports ∼60% PE for the inner membrane, closer to the E. coli “Native” PE composition. We hence keep both extracts as references (Table 1). Unlike the composition of the native extracts, that of the synthetic lipid mixtures is precisely controlled, allowing us to isolate the effects of specific lipids and acyl chain structures. This reduction in complexity enables more tractability for studies aiming to ascribe observations to specific molecular effects. In contrast, lipid extracts, while more biologically representative, often contain uncharacterized species and exhibit high heterogeneity.

Figure 1 displays the results from the DSC analysis, with our candidate samples graphically summarized in Figure S4A–C. In line with previous lipidomic studies (Table 1), E. coli Native and Polar Extract mixtures exhibit *T*_m_ = 22.7 ± 0.3 °C and *T*_m_ = 20.7 ± 0.4 °C, respectively. Mixtures containing POPG show a melting point significantly lower than those with DPPG, with an average 8–10 °C gap between equivalent mixtures. These differences reflect the *T*_m_ difference between the two pure lipid species and suggest their homogeneous mixing in POPE. The measured *T*_m_ values are in agreement with a previous work,^78^ where *T*_m_ of some of our binary mixtures have been previously tested, strengthening our DSC results. Moreover, the presence of a unique main peak in the DSC curves (see Figure S5) and its 20–40 kJ/mol change in enthalpy (see Figure S6) are also consistent with the melting transition of a homogeneous lipid membrane.^79^ All mixtures exhibit a *T*_m_ lower than 37 °C—our reference E. coli growth temperature. Ternary mixtures exhibit a more complex transition behavior, without any obvious trend for *T*_m_. This is expected from previous studies of CL-containing membranes.^80–82^

**Figure 1:**
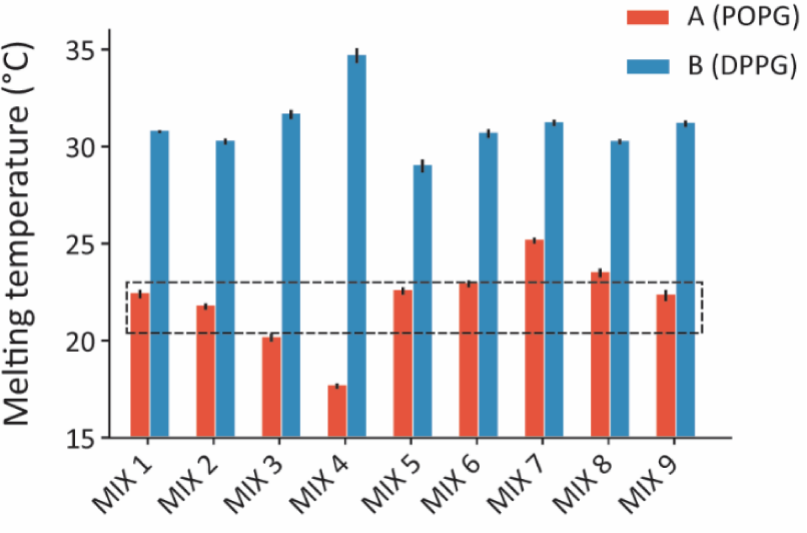
Melting temperatures of vesicles composed of the different model lipid mixtures aiming to replicate the behavior and lipid composition of the E. coli inner membrane grown at 37 °C. The dashed box encloses the values obtained for the native E. coli inner membrane in reference experiments. For each model system, the DSC results are given for POPG (red) and DPPG (blue) as a PG source. All the averages and standard deviations are presented in Table S1, where theoretical, measured, and corrected values are presented.

Comparing the results in Figure 1 to our reference E. coli Polar and Native Extract mixtures rules out the use of DPPG as a PG source for our model membrane. The best-matching candidates are three different ternary mixtures: 80% POPE, 15% POPG, and 5% CL (composition #5A); 75% POPE, 20% POPG, and 5% CL (composition #6A); and 65% POPE, 25% POPG, and 10% CL (composition #9A). Two binary mixtures also match E. coli Polar and Native Extract mixtures *T*_m_: 80% POPE and 20% POPG (composition #1A) and 75% POPE and 25% POPG (composition #2A). These mixtures still reflect the molecular ratios of the most abundant lipids reported for E. coli and might hence be sufficient for certain studies, provided CL is not critical.

Interestingly, if considering the recent report of only ∼60% PE content for the inner membrane,^59^ our results suggest a unique candidate for the E. coli model membrane: 65% POPE, 25% POPG, and 10% CL (composition #9-A).

### Mechanical Properties of the Candidate and E. coli Membranes

Before measuring the mechanical properties of the remaining candidate systems, it is necessary to demonstrate that the lipid mixtures can form stable and homogeneous unilamellar vesicles as well as smooth, stable supported lipid bilayers (SLBs). The formation of stable and homogeneous SLBs from SUV deposition is not obvious because CL can affect bilayer fluidity, evolution, and membrane packing,^83^ sometimes inducing molecular rearrangement over time and precluding the formation of stable flat SLBs.^66^ Additionally, the formation of SLBs with PE and PG has been previously reported as challenging due to the negative charge of the PG headgroups, the conformation of POPE/POPG molecules,^84^ and the effect of PE lipids on membrane curvature.^85^ Here, we employed one of our candidate mixtures (MIX 6A) to check the stability of model membrane systems containing PE/PG and CL. Stable and homogeneous SUVs were confirmed using optical microscopy (Figure 2A,B), showing rounded vesicles that remained stable for at least 14 days. Similarly, AFM imaging of candidate SLBs (Figure 2C) revealed smooth, stable patches. Here, SLBs were formed on an atomically flat mica surface using extruded 100 nm SUVs (see Materials and Methods). By working at a relatively low SUV concentration, isolated membrane patches could be formed and imaged, confirming the presence of a single, stable lipid bilayer in the fluid phase. The patch thickness of 4.7 ± 0.1 nm is in line with the expected thickness for such fluid bilayers^86^ (Figure 2D). Increasing the SUV concentration enabled full substrate coverage, with a membrane exhibiting only minor defects (see Figure S7).

**Figure 2:**
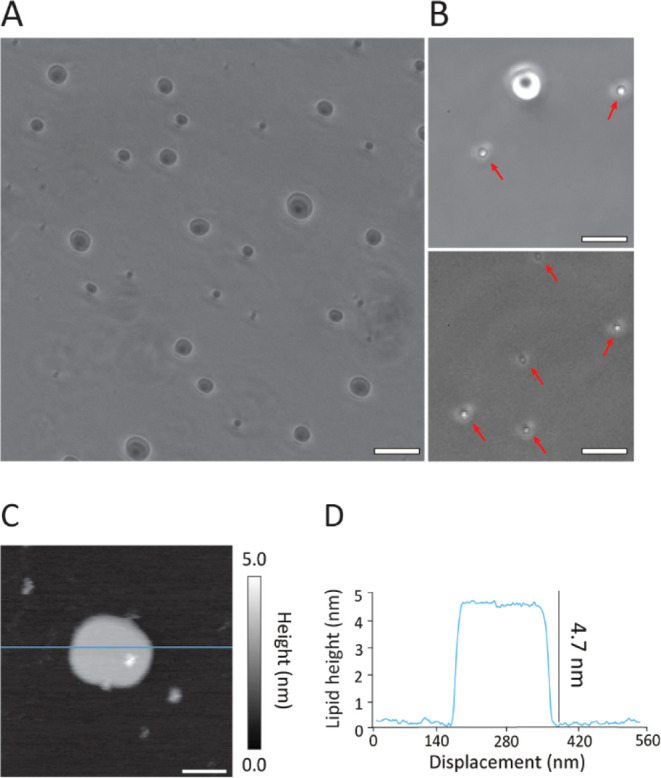
Demonstration of stable membrane formation with the candidate mixture 6A. In bulk solution (A,B), optical microscopy (phase contrast) shows the formation of stable vesicular model membrane systems for the ternary POPE–POPG–CL mixture. Images of spherical lipid vesicles formed with the mixture were taken with 10× (A) and 40× (B) objectives. ∼1 μm size vesicles have been indicated with red arrows for clarity (B). The vesicles were stable for 2 weeks after preparation. Stable SLBs could also be formed on a mica substrate in solution (C,D) with patches visible. AFM imaging (C) quantifies the thickness of a typical patch with an associated line profile (D). The scale bars are 50 μm (A—10× objective), 13 μm (B—40× objective), and 100 nm (C).

### Perpendicular Compression of the Membrane

The mechanical properties of the membranes, namely, *Y* and *K*_a_, are quantified from AFM nanoindentation measurements. For simplicity, we assume the membrane behaves as a homogeneous isotropic solid, in line with the single DSC peak. AFM can also distinguish between domains in different phases, ensuring membrane homogeneity on the scale of the measurement. Following this assumption, we use thin plate theory^87,88^ to relate *K*_a_ and *Y* through the well-known relationship:

3 $$K_{a}=\frac{Yh}{2\left(1-\nu \right)}$$

where *Y* is experimentally measured by AFM, *h* is the membrane thickness, and *v* is its Poisson ratio. AFM nanoindentation on SLBs requires correcting the established Hertz indentation model^89^ for the finite thickness of the membrane and the hard substrate underneath^48,52^ (see eq 2 in Materials and Methods and Figure S2 for more details). We probed the Young’s modulus of mixtures 6A and 9A and compared them with E. coli Polar Extract obtained in the same manner. E. coli Native is again given for completeness. The two lipid mixtures reflect model systems with different CL content (5% for 6A and 10% for 9A), known to shape the fluidity of the bilayer and, therefore, its mechanical properties. The mechanical assays were also performed on two negative controls: the DPPG-based mixture 2B and pure POPE, with both controls forming stable SLBs. In all cases, we distinguished the fluid pretransition phase (when cooling) and the more ordered post-transition phase when conducting the measurements (Figure 3). The more ordered domains are 0.5–0.7 nm taller than the remaining pretransition domains (see Figure S2A), consistent with the expected membrane thickness variation between the two phases.^90,91^ The relatively small differences between pre- and post-transition values for the E. coli samples and the MIX 6A and 9A come from the fact that both phases retain some level of molecular mobility (fluidity), albeit different; they represent different degrees of molecular ordering and should hence not be thought of as fluid and gel phases.

**Figure 3:**
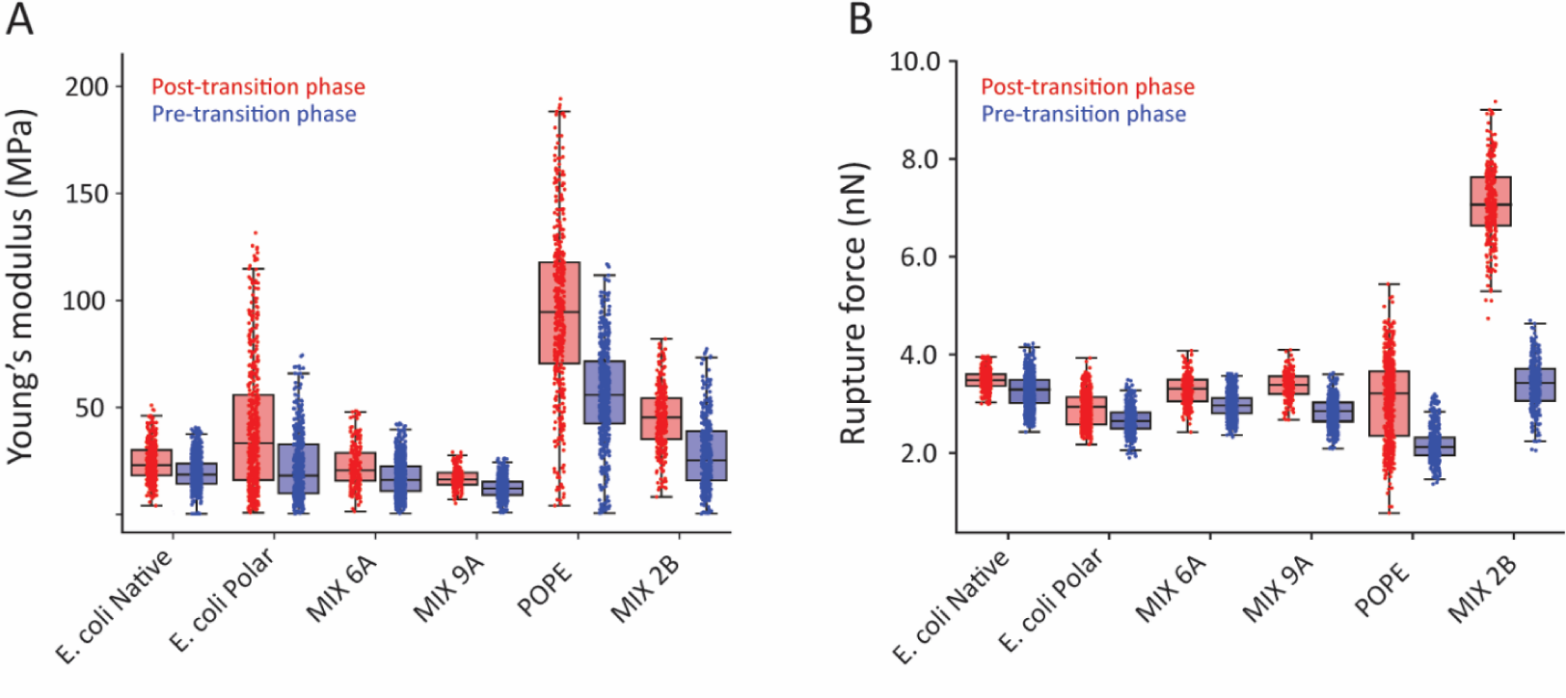
Analysis of the native and candidate model membranes using AFM nanomechanical indentation. (A) Membrane-averaged *Y* values, calculated from the indentation region of the curves (elastic indentation) and assuming a spherical tip (radius 12 nm, from the manufacturer). The results confirm the mechanical similarities between the E. coli references and the two ternary POPE–POPG–CL candidate mixtures. (B) When increasing the indentation force, the tip eventually punctures the membrane. The rupture force, *F*_r_, is an indication of the bilayer strength and cohesion. For each sample, all the force spectroscopy data have been acquired at 2 °C below the *T*_m_. For (A,B), the data is presented as boxplots distinguishing the post-transition and pretransition phases. The upper and lower whiskers extend to the furthest data point within 1.5 times the interquartile range, hence indicating the variability outside the upper and lower quartiles, respectively. The overlaid scatter plot shows the nanomechanical values for each single indentation performed on the membrane. Examples of force maps and measurement principles are illustrated in Figure S2B–D.

We performed so-called force maps^53,92^ whereby force-distance curves -the resistance experienced by the tip as it presses on the membrane- are systematically acquired across randomly selected regions of the membrane (see Figure S2C,D). From each curve, we immediately get *F*_r_ ^90^ and can calculate *Y*. While adhesion forces can also be probed using AFM information from the retraction segment of the force–distance curve, they are not included in our analysis. This is because our measurement involves the tip punching through the bilayer, making retraction features from the inside of the membrane difficult to interpret in terms of interactions between the tip and the surface of the membrane. However, no significant attractive features were observed in the approach curves, suggesting a low magnitude of adhesive interactions (Figure S8).

Both E. coli extracts and our two candidate mixtures show comparable values on the pretransition phase (*Y* ∼ 17.5 MPa and *F*_r_ ∼ 3.0 nN) and the more ordered post-transition phase (*Y* ∼ 25.8 MPa and *F*_r_ ∼ 3.4 nN). All of the averages and standard deviations are presented in Table S2. This similarity in mechanical properties is meaningful, as confirmed by the significantly different values obtained for the two negative controls (pure POPE and the DPPG-based samples).

### In-Plane Stretching/Compression of the Membrane

From the derived *Y* values and taking *v* = 0.5 (incompressible membrane) and *h* = 4.7 nm (obtained in Figure 2D), we can calculate *K*_a_ using eq 2. The results, given in Figure 4, suggest *K*_a_ values slightly lower than the average 0.2 N/m obtained with micropipette aspiration from previous reports on native E. coli spheroplasts and artificial lipid vesicles.^93–97^ A separate study on monolayers of mixtures similar to our 1A and 2A reports *K*_a_ values around 0.12 N/m,^27^ in line with our present results. The difference might be explained by AFM indentation measurements predominantly probing the top leaflet of the bilayer—effectively a monolayer. However, other sources of error may also be at play, including our assumption of the membrane behaving as a homogeneous isotropic solid. Still, both our out-of-plane and in-plane mechanical estimates support mixtures 6A and 9A as a suitable composition to reproduce the biophysical properties of E. coli’s inner membrane lipids.

**Figure 4:**
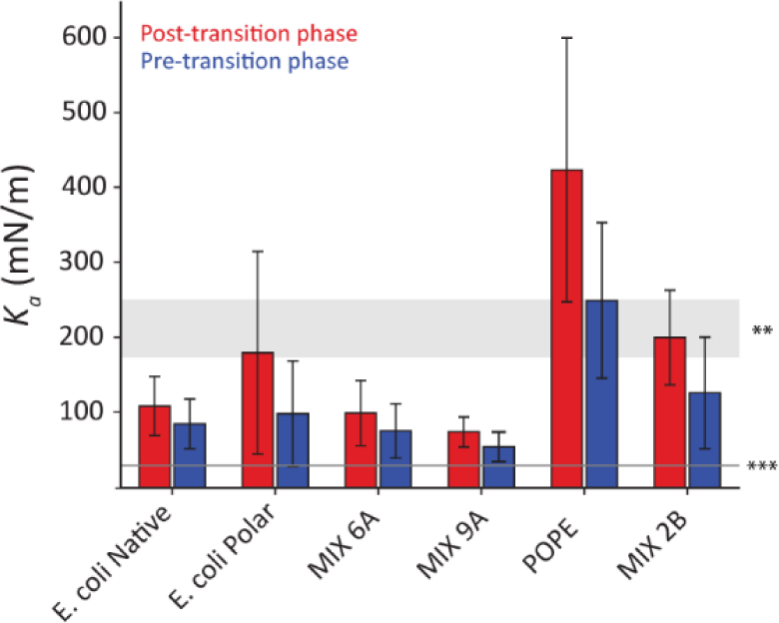
Comparison of the *K*_a_ values derived from the measured *Y* values through eq 3 for each phase of the different candidate membranes. The error bars represent the two standard deviations. *K*_a_ values reported in a previous study^98^ have been shown for comparison (light gray shaded areas), comprising values derived from artificial giant lipid vesicles** and metabolically active E. coli spheroplast***.

Overall, the results point to MIX 6A and 9A as suitable model systems that replicate the main aspects of E. coli’s inner membrane lipid composition and mechanical properties based on commercially available lipid mixtures. We find that DPPG is not suitable as a PG source for model E. coli membranes under standard growth conditions, although mixtures involving DPPG do not phase-separate despite their high *T*_m_ (single peaks in DSC, Figure S5). Instead, our results point to three ternary mixtures of POPE, POPG, and CL for model systems. Graphically comparing the composition of our model systems with previously reported E. coli membrane model mixtures^22,25–27,29,73,80,99–107^ (Figure S9) shows that most reported models differ substantially from each other and from our mixtures. Given the lack of any accepted standard models, the ability of our model systems to reproduce some of the main elements of the membranes’ biomechanical properties could be helpful for studies where active molecules or forces are at play, for example, investigations of force transduction within membranes or active response to stimuli achieved through integral proteins such as mechanosensitive channels and PIEZO proteins.^108–110^

The dynamical shift of *T*_m_ as a function of E. coli’s growth environment indicates a clear correlation between the membrane functionality, the activity of embedded macromolecules, and the overall physiological transduction of these stimuli across the cell. The model systems developed here could also be used to investigate the passive mechanisms behind lipid bilayer asymmetry, an intrinsic property of biological membranes,^21^ and its effects on the functional features of native membranes. Recent work highlighted how CL can show leaflet preferentiality depending on vesicle curvature,^111^ suggesting the possibility of developing model membrane systems with controlled compositional asymmetry that could be employed to explore this phenomenon in future work. More sophisticated models will be needed to account for the significant local variations in native membranes’ macromolecular content,^112^ an important element in many experimental^113,114^ and computational^115^ studies.

The consistency between the measurements on the two mechanically different lipid phases and the general agreement with existing literature support our present approach to estimate *K*_a_, but the results need to be taken with caution because the lipid bilayer is not an isotropic 3D material, as assumed with the thin plate model. Future studies aimed at determining the bending modulus of our model systems will likely be of interest, as well. For example, E. coli produces small spherical structures, termed bacterial vesicles, which help with the exchange of genetic material, molecules, and proteins.^118–120^ Presumably, these vesicles are under less pressure than E. coli, making the bending contribution to Helmholtz free energy relevant. One previous study used a mixture^107^ similar to our 8A model (with an 18:1 chain content set to 41:59, slightly higher than our 50:50) to study colistin but reported bending moduli of only single-component membranes.

## Conclusion

In this study, we identify minimal, lipid-only model systems for the E. coli inner membrane. The models, composed of synthetic lipids 16:0–18:1 phosphatidylethanolamine (POPE), 16:0–18:1 phosphatidylglycerol (POPG), and 16:0–18:1 cardiolipin (CL), achieve a similar lipid composition and ratio to that found in the native membrane and can reproduce its elastic moduli and thermomechanical properties. Like for all lipid-only model membranes, our model systems cannot fully reproduce the complexity of native membranes due to the absence of proteins, complex biomolecules, and a plethora of native lipids.^105,116,117^ Instead, our goal is to establish a simplified yet fully tractable system that captures key compositional and biophysical features of the E. coli inner membrane. We anticipate the models to be particularly useful for studies focusing on mechanical forces in E. coli membranes, for example, to investigate the function and mechano-transduction of membrane proteins or macromolecule–membrane interactions.

## Supplementary Material



## Data Availability

All the data presented in this paper are freely available at https://collections.durham.ac.uk/files/r22r36tx61b (doi:10.1101/2024.09.29.615671)

## Notes

Details of DSC and AFM measurement principles and derivation of the relevant parameters with additional data, table summary of the DSC and AFM results, and compositional comparison of our model membranes with models previously developed in the literature (PDF)

## References

[1] Watson H. (2015). Biological
Membranes. Essays Biochem..

[2] Hammond, C. Ionic Gradients, Membrane Potential and Ionic Currents. In Cellular and Molecular Neurophysiology; Elsevier, 2015, pp. 39–54.

[3] Rosko J., Martinez V. A., Poon W. C. K., Pilizota T. (2017). Osmotaxis in Escherichia Coli through Changes in Motor Speed. Proc. Natl.
Acad. Sci. U. S. A..

[4] Rosenbaum D. M., Rasmussen S. G. F., Kobilka B. K. (2009). The Structure and Function of G-Protein-Coupled
Receptors. Nature.

[5] Zhang Y., Daday C., Gu R. X., Cox C. D., Martinac B., de Groot B. L., Walz T. (2021). Visualization
of the Mechanosensitive
Ion Channel MscS under Membrane Tension. Nature.

[6] Vollmer W., Blanot D., De Pedro M. A. (2008). Peptidoglycan
Structure and Architecture. FEMS Microbiol.
Rev..

[7] Silhavy T. J., Kahne D., Walker S. (2010). The Bacterial Cell Envelope. Cold Spring Harbor Perspect. Biol..

[8] Cohen S. N., Chang A. C. Y., Boyer H. W., Helling R. B. (1973). Construction
of
Biologically Functional Bacterial Plasmids in Vitro. Proc. Natl. Acad. Sci. U. S. A..

[9] Cong L., Ran F. A., Cox D., Lin S., Barretto R., Habib N., Hsu P. D., Wu X., Jiang W., Marraffini L. A., Zhang F. (2013). Multiplex Genome Engineering
Using
CRISPR/Cas Systems. Science.

[10] Touchon M., Perrin A., de Sousa J. A. M., Vangchhia B., Burn S., O’Brien C. L., Denamur E., Gordon D., Rocha E. P. (2020). Phylogenetic Background
and Habitat Drive the Genetic
Diversification of Escherichia Coli. PloS Genet..

[11] Felden B., Paillard L. (2017). When Eukaryotes and
Prokaryotes Look Alike: The Case
of Regulatory RNAs. FEMS Microbiol. Rev..

[12] Sørensen H. P., Mortensen K. K. (2005). Advanced
Genetic Strategies for Recombinant Protein
Expression in Escherichia Coli. J. Biotechnol..

[13] Assenberg R., Wan P. T., Geisse S., Mayr L. M. (2013). Advances in Recombinant
Protein Expression for Use in Pharmaceutical Research. Curr. Op. Struct. Biol..

[14] Huang C. J., Lin H., Yang X. (2012). Industrial
Production of Recombinant Therapeutics in Escherichia
Coli and Its Recent Advancements. J. Industr. Microbiol. Biotechnol..

[15] Nakayama H., Mitsui T., Nishihara M., Kito M. (1980). Relation between Growth
Temperature of E. Coli and Phase Transition
Temperatures of Its Cytoplasmic and Outer Membranes. Biochim. Biophys. Acta, Biomembr..

[16] Morein S., Andersson A. S., Rilfors L., Lindblom G. (1996). Wild-Type Escherichia
Coli Cells Regulate the Membrane Lipid
Composition in a “Window” between Gel and Non-Lamellar
Structures. J. Biol. Chem..

[17] Mužić T., Tounsi F., Madsen S. B., Pollakowski D., Konrad M., Heimburg T. (2019). Melting Transitions
in Biomembranes. Biochim. Biophys. Acta, Biomembr..

[18] Sohlenkamp C., Geiger O. (2016). Bacterial Membrane
Lipids: Diversity in Structures
and Pathways. FEMS Microbiol. Rev..

[19] Carey A. B., Ashenden A., Köper I. (2022). Model Architectures
for Bacterial
Membranes. Biophys. Rev..

[20] Lugtenberg E. J. J., Peters R. (1976). Distribution of Lipids
in Cytoplasmic and Outer Membranes
of Escherichia Coli K12. Biochim. Biophys. Acta, Lipids Lipid Metab..

[21] Bogdanov M. (2023). Renovating
a Double Fence with or without Notifying the next Door and across
the Street Neighbors: Why the Biogenic Cytoplasmic Membrane of Gram-Negative
Bacteria Display Asymmetry?. Emerg. Top. Life
Sci..

[22] Hsieh M.-K., Klauda J. B. (2022). Leaflet Asymmetry Modeling in the Lipid Composition
of Escherichia Coli Cytoplasmic Membranes. J. Phys. Chem. B.

[23] Carranza G., Angius F., Ilioaia O., Solgadi A., Miroux B., Arechaga I. (2017). Cardiolipin Plays an Essential Role
in the Formation
of Intracellular Membranes in Escherichia Coli. Biochim. Biophys. Acta, Biomembr..

[24] Lopes S., Neves C. S., Eaton P., Gameiro P. (2010). Cardiolipin, a Key
Component to Mimic the E. Coli Bacterial
Membrane in Model Systems Revealed by Dynamic Light Scattering and
Steady-State Fluorescence Anisotropy. Anal.
Bioanal. Chem..

[25] Pandit K. R., Klauda J. B. (2012). Membrane Models of E. Coli Containing Cyclic Moieties in the Aliphatic
Lipid Chain. Biochim. Biophys. Acta, Biomembr..

[26] Shearer J., Marzinek J. K., Bond P. J., Khalid S. (2020). Molecular Dynamics
Simulations of Bacterial Outer Membrane Lipid Extraction: Adequate
Sampling?. J. Chem. Phys..

[27] Hwang H., Paracini N., Parks J. M., Lakey J. H., Gumbart J. C. (2018). Distribution
of Mechanical Stress in the Escherichia Coli Cell Envelope. Biochim. Biophys. Acta, Biomembr..

[28] Piggot T. J., Holdbrook D. A., Khalid S. (2013). Conformational Dynamics and Membrane
Interactions of the E. Coli Outer Membrane
Protein FecA: A Molecular Dynamics Simulation Study. Biochim. Biophys. Acta, Biomembr..

[29] Murzyn K., Róg T., Pasenkiewicz-Gierula M. (2005). Phosphatidylethanolamine-Phosphatidylglycerol
Bilayer as a Model of the Inner Bacterial Membrane. Biophys. J..

[30] Li S., Ren R., Lyu L., Song J., Wang Y., Lin T.-W., Brun A. L., Hsu H.-Y., Shen H.-H. (2022). Solid and
Liquid
Surface-Supported Bacterial Membrane Mimetics as a Platform for the
Functional and Structural Studies of Antimicrobials. Membranes.

[31] Bogdanov M., Mileykovskaya E., Dowhan W. (2008). Lipids in the Assembly of Membrane
Proteins and Organization of Protein Supercomplexes: Implications
for Lipid-Linked Disorders. Biochem. Soc. Trans..

[32] Lingwood D., Simons K. (2010). Lipid Rafts as a Membrane-Organizing
Principle. Science.

[33] Chan Y.-H. M., Boxer S. G. (2007). Model Membrane Systems
and Their Applications State
of the Field. Curr. Opin. Chem. Biol..

[34] Eeman M., Deleu M. (2010). From Biological Membranes
to Biomimetic Model Membranes. Biotechnol. Agron.
Soc. Environ..

[35] Shokri A., Larsson G. (2004). Characterisation of
the Escherichia
Coli Membrane Structure and Function during Fedbatch
Cultivation. Microb. Cell Fact..

[36] Oliver P. M., Crooks J. A., Leidl M., Yoon E. J., Saghatelian A., Weibel D. B. (2014). Localization of Anionic Phospholipids in Escherichia Coli Cells. J.
Bacteriol..

[37] Cevc G. (1991). How Membrane
Chain-Melting Phase-Transition Temperature Is Affected by the Lipid
Chain Asymmetry and Degree of Unsaturation: An Effective Chain-Length
Model. Biochemistry.

[38] Wong F., Amir A. (2019). Mechanics and Dynamics
of Bacterial Cell Lysis. Biophys. J..

[39] Safran, S. A. Statistical Thermodynamics of Surfaces, Interfaces, and Membranes; CRC Press, 2018.

[40] Han M. J. (2016). Exploring
the Proteomic Characteristics of the Escherichia Coli B and K-12 Strains in Different Cellular Compartments. J. Biosci. Bioengin..

[41] Yoon S. H., Han M.-J., Jeong H., Lee C. H., Xia X.-X., Lee D.-H., Shim J. H., Lee S. Y., Oh T. K., Kim J. F. (2012). Comparative Multi-Omics Systems Analysis
of Escherichia Coli Strains B and K-12. Genome. Biol..

[42] Baccouch R., Shi Y., Vernay E., Mathelié-Guinlet M., Taib-Maamar N., Villette S., Feuillie C., Rascol E., Nuss P., Lecomte S., Molinari M., Staneva G., Alves I. D. (2023). The Impact
of Lipid Polyunsaturation on the Physical and Mechanical Properties
of Lipid Membranes. Biochim. Biophys. Acta -
Biomembr..

[43] Giuliano C. B., Cvjetan N., Ayache J., Walde P. (2021). Multivesicular Vesicles:
Preparation and Applications. ChemSystemschem.

[44] Toda A., Hikosaka M., Yamada K. (2002). Superheating of the
Melting Kinetics
in Polymer Crystals: A Possible Nucleation Mechanism. Polymer.

[45] Toda A. (2016). Heating Rate
Dependence of Melting Peak Temperature Examined by DSC of Heat Flux
Type. J. Therm. Anal. Calorim..

[46] Krasnopeeva E., Lo C. J., Pilizota T. (2019). Single-Cell
Bacterial Electrophysiology
Reveals Mechanisms of Stress-Induced Damage. Biophys. J..

[47] Butt H. J., Jaschke M. (1995). Calculation of Thermal Noise in Atomic Force Microscopy. Nanotechnology.

[48] Garcia P. D., Garcia R. (2018). Determination of the Elastic Moduli
of a Single Cell
Cultured on a Rigid Support by Force Microscopy. Biophys. J..

[49] Voïtchovsky K., Contera S. A., Kamihira M., Watts A., Ryan J. F. (2006). Differential
Stiffness and Lipid Mobility in the Leaflets of Purple Membranes. Biophys. J..

[50] Geissler E., Hecht A. M. (1981). The Poisson Ratio
in Polymer Gels. 2. Macromolecules.

[51] Mahaffy R. E., Shih C. K., MacKintosh F. C., Käs J. (2000). Scanning Probe-Based
Frequency-Dependent Microrheology of Polymer Gels and Biological Cells. Phys. Rev. Lett..

[52] Dimitriadis E. K., Horkay F., Maresca J., Kachar B., Chadwick R. S. (2002). Determination
of Elastic Moduli of Thin Layers of Soft Material Using the Atomic
Force Microscope. Biophys. J..

[53] Picas L., Rico F., Scheuring S. (2012). Direct Measurement
of the Mechanical
Properties of Lipid Phases in Supported Bilayers. Biophys. J..

[54] Gabbutt C., Shen W., Seifert J., Contera S. (2019). AFM Nanoindentation
Reveals Decrease of Elastic Modulus of Lipid Bilayers near Freezing
Point of Water. Sci. Rep..

[55] Nečas D., Klapetek P. (2012). Gwyddion: An Open-Source Software
for SPM Data Analysis. Open Phys..

[56] Schindelin J., Arganda-Carreras I., Frise E., Kaynig V., Longair M., Pietzsch T., Preibisch S., Rueden C., Saalfeld S., Schmid B., Tinevez J. Y., White D. J., Hartenstein V., Eliceiri K., Tomancak P., Cardona A. (2012). Fiji: An Open-Source
Platform for Biological-Image Analysis. Nat.
Methods.

[57] Pritchard L., White J. A., Birch P. R. J., Toth I. K. (2006). GenomeDiagram: A
Python Package for the Visualization of Large-Scale Genomic Data. Bioinformatics.

[58] Jackson M. B., Cronan J. E. (1978). An Estimate of the Minimum Amount
of Fluid Lipid Required
for the Growth of Escherichia Coli. Biochim. Biophys. Acta, Protein Struct. Mol. Enzymol..

[59] Bogdanov M., Pyrshev K., Yesylevskyy S., Ryabichko S., Boiko V., Ivanchenko P., Kiyamova R., Guan Z., Ramseyer C., Dowhan W. (2020). Phospholipid
Distribution in the
Cytoplasmic Membrane of Gram-Negative Bacteria Is Highly Asymmetric,
Dynamic, and Cell Shape-Dependent. Sci. Adv..

[60] Casadei M. A., Mañas P., Niven G., Needs E., Mackey B. M. (2002). Role of
Membrane Fluidity in Pressure Resistance of Escherichia
Coli NCTC 8164. Appl. Environ.
Microbiol..

[61] Ridone P., Nakayama Y., Martinac B., Battle A. R. (2015). Patch Clamp Characterization
of the Effect of Cardiolipin on MscS of E. Coli.
Eur. Biophys. J..

[62] Corey R. A., Song W., Duncan A. L., Ansell T. B., Sansom M. S. P., Stansfeld P. J. (2021). Identification and Assessment of
Cardiolipin Interactions
with E. Coli Inner Membrane Proteins. Sci. Adv..

[63] Lopes S. C., Ivanova G., de Castro B., Gameiro P. (2018). Revealing Cardiolipins
Influence in the Construction of a Significant Mitochondrial Membrane
Model. Biochim. Biophys. Acta, Protein Struct.
Mol. Enzymol..

[64] Oursel D., Loutelier-Bourhis C., Orange N., Chevalier S., Norris V., Lange C. M. (2007). Lipid Composition of Membranes of Escherichia Coli by Liquid Chromatography/Tandem
Mass Spectrometry Using Negative Electrospray Ionization. Rapid Commun. Mass Spectrom..

[65] Maiti A., Kumar A., Daschakraborty S. (2023). How Do Cyclopropane
Fatty Acids Protect
the Cell Membrane of Escherichia coli in Cold Shock?. J. Phys. Chem. B.

[66] Unsay J. D., Cosentino K., Subburaj Y., García-Sáez A. J. (2013). Cardiolipin
Effects on Membrane Structure and Dynamics. Langmuir.

[67] Mitchison-Field L. M., Belin B. J. (2023). Bacterial
lipid biophysics and membrane organization. Curr. Opin. Microbiol..

[68] Kannenberg E. L., Poralla K. (1999). Hopanoid Biosynthesis and Function in Bacteria. Naturwissenschaften.

[69] Renner L. D., Weibel D. B. (2011). Cardiolipin microdomains localize to negatively curved
regions of Escherichia coli membranes. Proc. Natl. Acad. Sci. U. S. A..

[70] Neidhardt F. C., Bloch P. L., Smith D. F. (1974). Culture
Medium for Enterobacteria. J. Bacteriol..

[71] Scott M., Gunderson C. W., Mateescu E. M., Zhang Z., Hwa T. (2010). Interdependence
of Cell Growth. Science.

[72] Honda T., Cremer J., Mancini L., Zhang Z., Pilizota T., Hwa T. (2022). Coordination of Gene
Expression with Cell Size Enables Escherichia Coli to Efficiently Maintain Motility
across Conditions. Proc. Natl. Acad. Sci. U.
S. A..

[73] Biltonen R. L., Lichtenberg D. (1993). The Use of Differential Scanning
Calorimetry as a Tool
to Characterize Liposome Preparations. Chem.
Phys. Lipids.

[74] Okotrub K. A., Zaytseva I. V., Adichtchev S. V., Surovtsev N. V. (2021). Raman Spectroscopy
and DSC Assay of the Phase Coexistence in Binary DMPC/Cholesterol
Multilamellar Vesicles. Biochim. Biophys. Acta,
Biomembr..

[75] Drazenovic J., Wang H., Roth K., Zhang J., Ahmed S., Chen Y., Bothun G., Wunder S. L. (2015). Effect of Lamellarity
and Size on Calorimetric Phase Transitions in Single Component Phosphatidylcholine
Vesicles. Biochim. Biophys. Acta, Biomembr..

[76] Mcelhaney R. N. (1982). The Use
of Differential Scanning Calorimetry and Differential Thermal Analysis
in Studies of Model and Biological Membranes. Chem. Phys. Lipids.

[77] Chiu M., Prenner E. (2011). Differential Scanning Calorimetry:
An Invaluable Tool
for a Detailed Thermodynamic Characterization of Macromolecules and
Their Interactions. J. Pharm. Bioallied Sci..

[78] Lopes S. C., Neves C. S., Eaton P., Gameiro P. (2012). Improved Model Systems
for Bacterial Membranes from Differing Species: The Importance of
Varying Composition in PE/PG/Cardiolipin Ternary Mixtures. Mol. Membr. Biol..

[79] Peters T. J. (1988). Cellular
Biology of Ectoenzymes. Cell Biochem. Funct..

[80] Lewis R. N. A. H., McElhaney R. N. (2009). The Physicochemical
Properties of Cardiolipin Bilayers
and Cardiolipin-Containing Lipid Membranes. Biochim. Biophys. Acta, Biomembr..

[81] Nichols-Smith S., Teh S. Y., Kuhl T. L. (2004). Thermodynamic
and Mechanical Properties
of Model Mitochondrial Membranes. Biochim. Biophys.
Acta, Biomembr..

[82] Domènech Ò., Sanz F., Montero M. T., Hernández-Borrell J. (2006). Thermodynamic
and Structural Study of the Main Phospholipid Components Comprising
the Mitochondrial Inner Membrane. Biochim. Biophys.
Acta, Biomembr..

[83] Wilson B. A., Ramanathan A., Lopez C. F. (2019). Cardiolipin-Dependent
Properties
of Model Mitochondrial Membranes from Molecular Simulations. Biophys. J..

[84] Lind T. K., Skoda M. W. A., Cárdenas M. (2019). Formation
and Characterization of
Supported Lipid Bilayers Composed of Phosphatidylethanolamine and
Phosphatidylglycerol by Vesicle Fusion, a Simple but Relevant Model
for Bacterial Membranes. ACS Omega.

[85] McMahon H. T., Boucrot E. (2015). Membrane Curvature
at a Glance. J. Cell Sci..

[86] Regan D., Williams J., Borri P., Langbein W. (2019). Lipid Bilayer Thickness
Measured by Quantitative DIC Reveals Phase Transitions and Effects
of Substrate Hydrophilicity. Langmuir.

[87] Lifshitz, E. M. ; Kosevich, A. M. ; Pitaevskii, L. P. Theory of Elasticity, 3rd ed.; Elsevier, 1986.

[88] Deserno M. (2015). Fluid Lipid
Membranes: From Differential Geometry to Curvature Stresses. Chem. Phys. Lipids.

[89] Hertz H. (1882). Ueber Die
Berührung Fester Elastischer Körper. J. Reine Angew. Math..

[90] Unsay J. D., Cosentino K., García-Sáez A. J. (2015). Atomic Force Microscopy
Imaging and Force Spectroscopy of Supported Lipid Bilayers. J. Vis. Exp.

[91] Alessandrini A., Facci P. (2014). Phase Transitions in
Supported Lipid Bilayers Studied by AFM. Soft
Matter.

[92] Garcia-Manyes S., Sanz F. (2010). Nanomechanics
of Lipid Bilayers by Force Spectroscopy with AFM: A
Perspective. Biochim. Biophys. Acta, Biomembr..

[93] Evans E., Heinrich V., Ludwig F., Rawicz W. (2003). Dynamic Tension Spectroscopy
and Strength of Biomembranes. Biophys. J..

[94] Rutkowski C. A., Williams L. M., Haines T. H., Cummins H. Z. (1991). The Elasticity of
Synthetic Phospholipid Vesicles Obtained by Photon Correlation Spectroscopy. Biochemistry.

[95] Rawicz W., Olbrich K. C., McIntosh T., Needham D., Evans E. A. (2000). Effect
of Chain Length and Unsaturation on Elasticity of Lipid Bilayers. Biophys. J..

[96] Sun S. T., Milon A., Tanaka T., Ourisson G., Nakatani Y. (1986). Osmotic Swelling
of Unilamellar Vesicles by the Stopped-Flow Light Scattering Method.
Elastic Properties of Vesicles. Biochim. Biophys.
Acta, Biomembr..

[97] Hantz E., Cao A., Escaig J., Taillandier E. (1986). The Osmotic
Response of Large Unilamellar
Vesicles Studied by Quasielastic Light Scattering. Biochim. Biophys. Acta, Biomembr..

[98] Sun Y., Sun T. L., Huang H. W. (2014). Physical
Properties of Escherichia Coli Spheroplast
Membranes. Biophys. J..

[99] Seeger H. M., Marino G., Alessandrini A., Facci P. (2009). Effect of Physical
Parameters on the Main Phase Transition of Supported Lipid Bilayers. Biophys. J..

[100] Mukherjee S., Kar R. K., Nanga R. P. R., Mroue K. H., Ramamoorthy A., Bhunia A. (2017). Accelerated Molecular Dynamics Simulation
Analysis of MSI-594 in a Lipid Bilayer. Phys.
Chem. Chem. Phys..

[101] Luchini A., Cavasso D., Radulescu A., D’Errico G., Paduano L., Vitiello G. (2021). Structural Organization
of Cardiolipin-Containing Vesicles as Models of the Bacterial Cytoplasmic
Membrane. Langmuir.

[102] Picas L., Montero M. T., Morros A., Oncins G., Hernández-Borrell J. (2008). Phase Changes in Supported
Planar
Bilayers of 1-Palmitoyl-2-Oleoyl- *Sn* -Glycero-3-Phosphoethanolamine. J. Phys. Chem. B.

[103] Boge L., Browning K. L., Nordström R., Campana M., Damgaard L. S. E., Seth Caous J., Hellsing M., Ringstad L., Andersson M. (2019). Peptide-Loaded
Cubosomes Functioning as an Antimicrobial Unit against Escherichia Coli. ACS Appl.
Mater. Interfaces.

[104] Cetuk H., Maramba J., Britt M., Scott A. J., Ernst R. K., Mihailescu M., Cotten M. L., Sukharev S. (2020). Differential
Interactions of Piscidins with Phospholipids and Lipopolysaccharides
at Membrane Interfaces. Langmuir.

[105] Pluhackova K., Horner A. (2021). Native-like Membrane
Models of E. Coli Polar Lipid Extract
Shed Light on the Importance
of Lipid Composition Complexity. BMC Biol..

[106] Wydro P. (2013). The Influence of Cardiolipin on Phosphatidylglycerol/Phosphatidylethanolamine
Monolayers-Studies on Ternary Films Imitating Bacterial Membranes. Coll. Surf. B.

[107] Dupuy F. G., Pagano I., Andenoro K., Peralta M. F., Elhady Y., Heinrich F., Tristram-Nagle S. (2018). Selective
Interaction of Colistin with Lipid Model Membranes. Biophys. J..

[108] Jin P., Jan L. Y., Jan Y. N. (2020). Mechanosensitive
Ion Channels: Structural
Features Relevant to Mechanotransduction Mechanisms. Annu. Rev. Neurosci..

[109] Rasmussen T., Rasmussen A., Yang L., Kaul C., Black S., Galbiati H., Conway S. J., Miller S., Blount P., Booth I. R. (2019). Interaction
of the Mechanosensitive
Channel, MscS, with the Membrane Bilayer through Lipid Intercalation
into Grooves and Pockets. J. Mol. Biol..

[110] Haswell E. S., Phillips R., Rees D. C. (2011). Mechanosensitive
Channels: What Can They Do and How Do They Do It?. Structure.

[111] Elmer-Dixon M. M., Hoody J., Steele H. B. B., Becht D. C., Bowler B. E. (2019). Cardiolipin Preferentially Partitions
to the Inner
Leaflet of Mixed Lipid Large Unilamellar Vesicles. J. Phys. Chem. B.

[112] Harayama T., Riezman H. (2018). Understanding the Diversity of Membrane
Lipid Composition. Nat. Rev. Mol. Cell Biol..

[113] Uray I. P., Uray K. (2021). Mechanotransduction
at the Plasma
Membrane-Cytoskeleton Interface. Int. J. Mol.
Sci..

[114] Barrett D. W., John R. K., Thrasivoulou C., Mata A., Deprest J. A., Becker D. L., David A. L., Chowdhury T. T. (2019). Targeting Mechanotransduction Mechanisms and Tissue
Weakening Signals in the Human Amniotic Membrane. Sci. Rep..

[115] Pliotas C., Dahl A. C. E., Rasmussen T., Mahendran K. R., Smith T. K., Marius P., Gault J., Banda T., Rasmussen A., Miller S., Robinson C. V., Bayley H., Sansom M. S. P., Booth I. R., Naismith J. H. (2015). The Role
of Lipids in Mechanosensation. Nat. Struct.
Mol. Biol..

[116] Ingólfsson H. I., Bhatia H., Zeppelin T., Bennett W. F. D., Carpenter K. A., Hsu P.-C., Dharuman G., Bremer P.-T., Schiott B., Lightstone F. C. (2020). Capturing Biologically
Complex Tissue Specific Membranes at Different Levels of Compositional
Complexity. J. Phys. Chem. B.

[117] Gazerani G., Piercey L. R., Reema S., Wilson K. A. (2025). Examining
the Biophysical Properties of the Inner Membrane of Gram-Negative
ESKAPE Pathogens. J. Chem. Inf. Model..

[118] Bitto N. J., Chapman R., Pidot S., Costin A., Lo C., Choi J., D’Cruze T., Reynolds E. C., Dashper S. G., Turnbull L. (2017). Bacterial
Membrane Vesicles Transport Their
DNA Cargo into Host Cells. Sci. Rep..

[119] Toyofuku M. (2019). Bacterial Communication through Membrane
Vesicles. Biosci., Biotechnol., Biochem..

[120] Mashburn L. M., Whiteley M. (2005). Membrane Vesicles Traffic
Signals
and Facilitate Group Activities in a Prokaryote. Nature.

