# Supplementary material for: A Minimalist Model Lipid System Mimicking the Biophysical Properties of Escherichia coli’s Inner Membrane
Source: Langmuir. 2025 May 7;41(19):12301–10. doi: 10.1021/acs.langmuir.5c01138 (PMC12100707; doi:10.1021/acs.langmuir.5c01138)
Supplement: Supplementary file 1 [file la5c01138_si_001.pdf]

Supporting Information

A minimalist model lipid system mimicking the biophysical properties of *Escherichia coli's* inner membrane

Nicolo Tormena[1], Teuta Pilizota[*,2,3], Kislon Voitchovsky[*,1]

1. Physics Department, Durham University, South Road, Durham DH1 3LE, UK
2. School of Biological Sciences and Centre for Engineering Biology, The University of Edinburgh, Alexander Crum Brown Road, Edinburgh, EH9 3FF, UK
3. Department of Physics, University of Cambridge, JJ Thompson Avenue, CB3 0HE, Cambridge, UK

*Correspondance: tp579@cam.ac.uk, kislon.voitchovsky@durham.ac.uk

Content of the Supporting Information (chronologically)**:**

- **Figure S1**: DSC thermographs of LMVs solution and optimization of experimental parameters.
- **Figure S2**: measurement principles for AFM force spectroscopy and force maps.
- **Figure S3**: Schematic representation of the lipids used in this work.
- **Table S1**: Summary the results obtained from the DSC calorimetric analysis performed on the LMVs including theoretical, experimental and corrected T_m values for all the analysed mixtures.
- **Figure S4**: Ternary diagram representations of the different mixtures analysed and their respective melting point.
- **Figure S5**: DSC thermographs of LMVs solution made of mixtures based on POPG and DPPG.
- **Figure S6**: Average melting enthalpy change from DSC experiments on LMVs solutions.
- **Figure S7**: AFM experiments proving the stability of SLBs from a model ternary mixture.
- **Table S2.** Summary the results obtained from the AFM force maps on the different lipid mixtures.
- **Figure S8**: Examples of AFM force curves showing no adhesion between the tip and SLB's headgroups.
- **Figure S9**: Compositional comparison of our model membranes with models in the literature.
- **References**

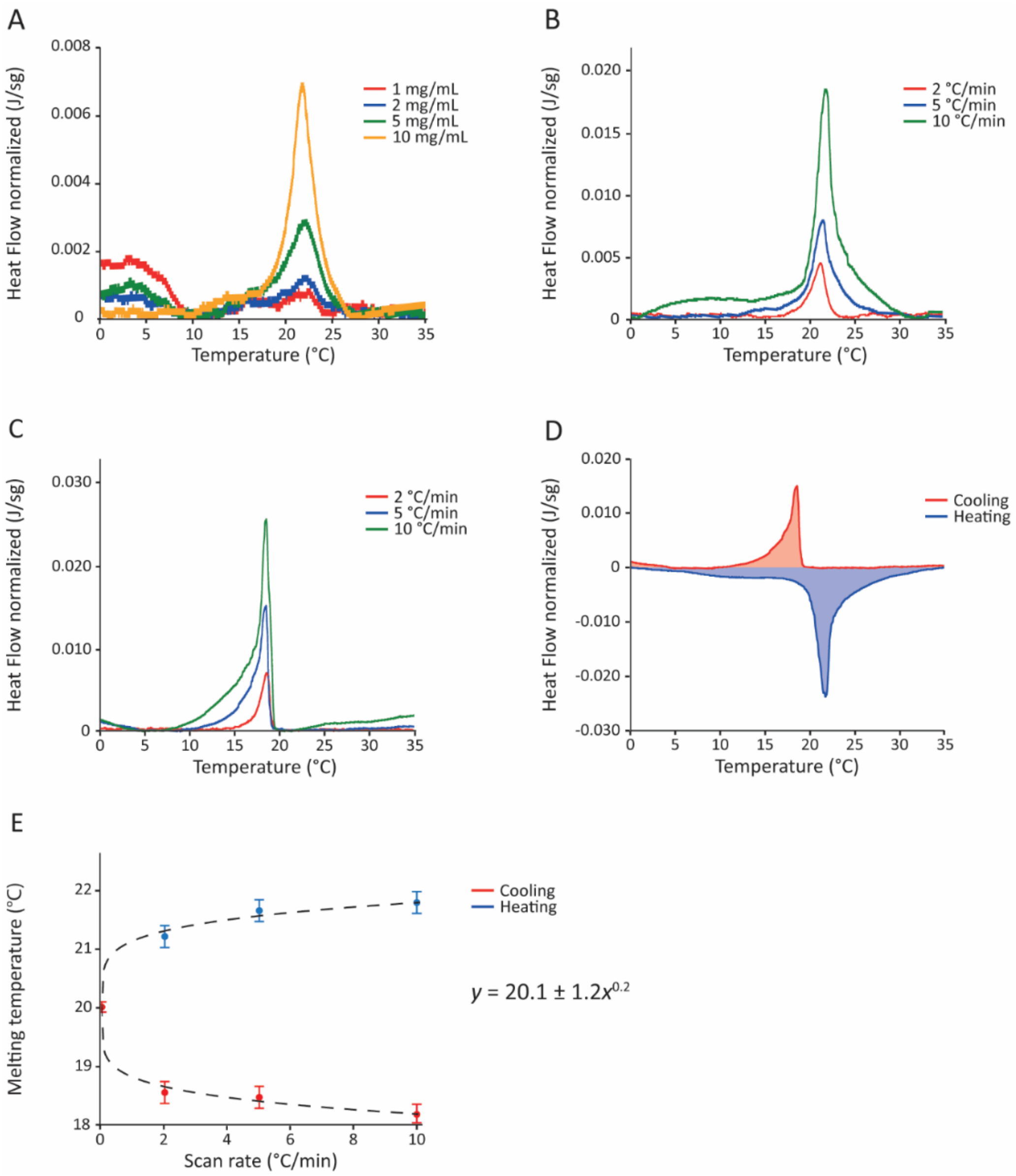

Figure S1: DSC thermographs of the LMVs solution used to optimize the DSC parameters. Normalised heat flow obtained from DSC experiments with varying (A) LMVs concentration at a scan rate of 5°C/min and (B) heating rate with 10 mg/m LMVs concentration. The measurements are performed to identify the optimal signal to noise ratio (SNR). Variation of the scan rates up to 10 °C/min leads to small calorimetric differences (melting point variations up to 0.4 °C). The same effects are detected on both (B) heating and (C) cooling DSC thermographs (both with 10 mg/mL LMVs concentration). (D) Direct comparison between the cooling and heating DSC experiments using a rate of 5 °C/min (10 mg/mL LMVs concentration). By using optimal concentration and heating rate, thermograph distortion is limited with relatively small variation of sample's thermodynamic parameters such as the melting point (<3 °C), while still retaining a significant SNR. (E) The melting temperature dependence on scan rate can be used to infer the equilibrium transition temperature (i.e. rate of zero). The nonlinear dependence of the melting temperature with the scan rate has been previously described [1,2] as $T_{m,\beta} = T_m + B\beta^z$, where $T_{m,\beta}$ is the measured melting temperature at each scan rate, T_m is the equilibrium or 'true' melting temperature, β is the scan rate, and B and z are fitting parameters. Here, B = 1.2 and z = 0.2. Error bars represent two standard deviations calculated from 3 different DSC runs per rate.

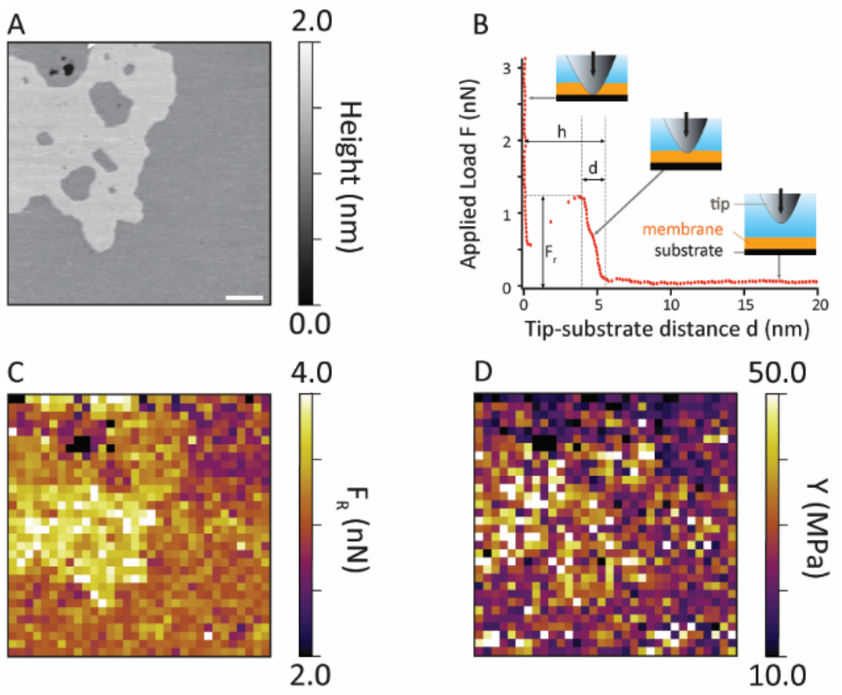

Figure S2: AFM force spectroscopy and force map measurement principles. (A) AFM topographical image of the ternary mixture (MIX – 6A) below its melting point. Two lipid phases, pre-transition the more ordered post-transition, appear darker and lighter grey respectively. (B) Example of a force spectroscopy curve acquired on a lipid bilayer with cartoons representing the different stages of the measurement process. Briefly, when the AFM tip approaches the membrane from the solution, the applied load is zero, unless adhesion is present (appearing as a negative load). Upon contacting the membrane, the applied load begins to increase, compressing and indenting the membrane over a depth d, until the applied load is strong enough to puncture through the bilayer (force F_r) and rest on the substrate underneath. From the analysis of the load – tip distance curves, it is possible to estimate the membrane Young's Modulus, Y, by fitting the experimental indentation curve with a suitable model (discussed in the Material and Methods section) [3,4]. The total distance $h \sim 5$ nm between the point of contact and the substrate corresponds to the thickness of the indented layer, confirming the presence of a single bilayer. (C-D) Force maps performed over the lipid membrane shown in (A) are used to extract F_r and Y. The scale bar in (A) is 200 nm.

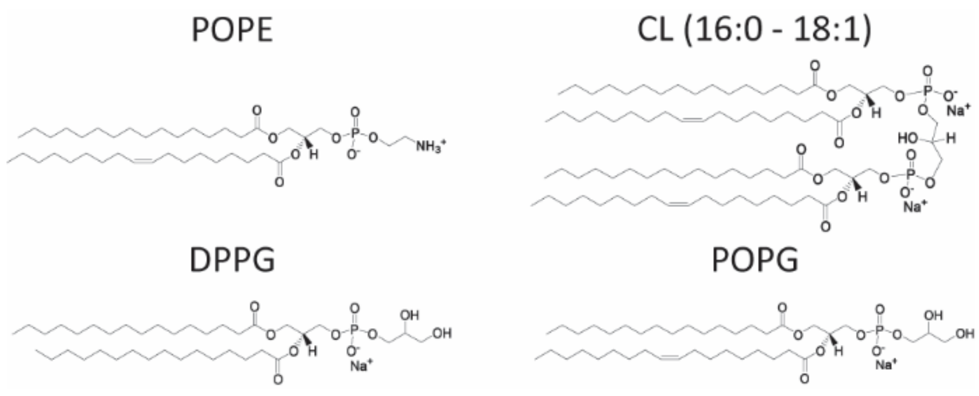

Figure S3: Schematic Lewis structures of the lipids used to produce the candidate mixtures. Standard abbreviations are used for the different lipid names. The molecular structures were reproduced from the information provided by Avanti Polar Lipids, the company from which the different lipids were purchased.

Mixture name	Predicted T_m (°C)	*Experimental* T_m (°C)	*Corrected* T_m (°C)
**1-A**	**19.6**	***22.4 ± 0.2***	***20.7 ± 0.2***
1-B	28.2	*30.8 ± 0.1*	*29.1 ± 0.1*
**2-A**	18.3	***21.8 ± 0.2***	***20.0 ± 0.2***
2-B	29	*30.3 ± 0.2*	*28.6 ± 0.2*
3-A	16.9	*20.1 ± 0.2*	*18.4 ± 0.2*
3-B	29.8	*31.7 ± 0.2*	*30.0 ± 0.2*
4-A	14.2	*17.7 ± 0.1*	*16.0 ± 0.1*
4-B	31.4	*34.7 ± 0.4*	*33.0 ± 0.4*
**5-A**	**-**	***22.6 ± 0.2***	***20.9 ± 0.2***
5-B	-	*29.0 ± 0.3*	*27.3 ± 0.3*
**6-A**	**-**	***22.9 ± 0.2***	***21.2 ± 0.2***
6-B	-	*30.7 ± 0.2*	*29.0 ± 0.2*
7-A	-	*25.2 ± 0.2*	*23.5 ± 0.2*
7-B	-	*31.2 ± 0.2*	*29.5 ± 0.2*
8-A	-	*23.5 ± 0.2*	*21.8 ± 0.2*
8-B	-	*30.3 ± 0.1*	*28.6 ± 0.1*
**9-A**	**-**	***22.3 ± 0.3***	***20.6 ± 0.3***
9-B	-	*31.2 ± 0.2*	*29.5 ± 0.2*
***E. coli* Native**	**-**	***22.7 ± 0.3***	***21.0 ± 0.3***
***E. coli* P. Extract**	**-**	***20.7 ± 0.4***	***19.0 ± 0.4***

Table S1: Candidate lipid mixtures with the T_m theoretically calculated and experimentally measured, with and without considering DSC scan rate effects. The reported experimental T_m are obtained through DSC experiments, and the correction factor in each case was calculated as described in Fig. S1 E. The mixtures highlighted better match the transition temperature of native *E. coli* extracts. The theoretical T_m have been calculated using the weighted arithmetic mean [5] to help identify a suitable temperature range for the measurements. Not all the theoretical values were calculated since, to the best of our knowledge, no information regarding the melting of pure CL is available.

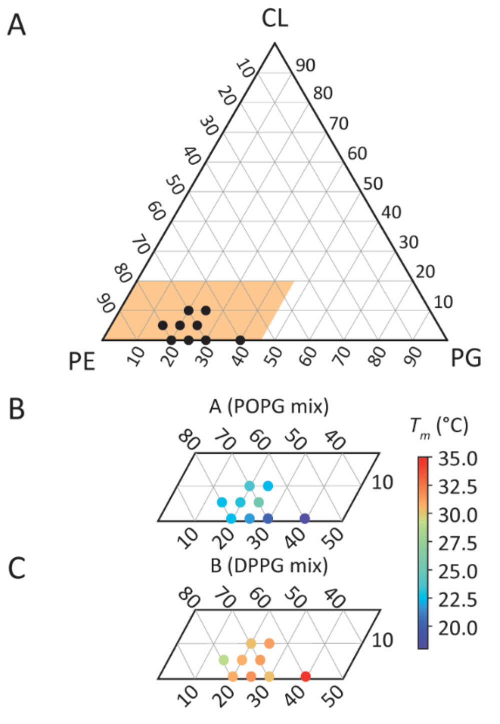

Figure S4: Graphical representation of the candidate mixtures measured with DSC with their respective T_m. (A) Ternary diagrams of the PE-PG-CL model systems explored showing the physiological composition range of *E. coli* 's inner membrane. (B-C) Magnified view of the ternary diagrams of the (B) POPG- and (C) DPPG-based mixtures together with their T_m (see Materials and Methods for details).

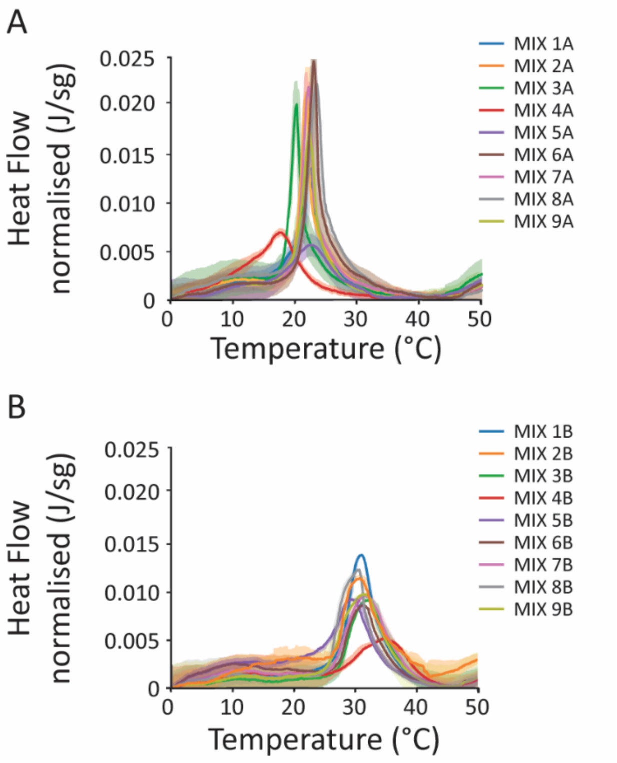

Figure S5: DSC thermographs of LMVs solution made of (A) POPG- and (B) DPPG-based mixtures. The average curve for each mixture is shown with its standard deviation (semi-transparent areas around the curves).

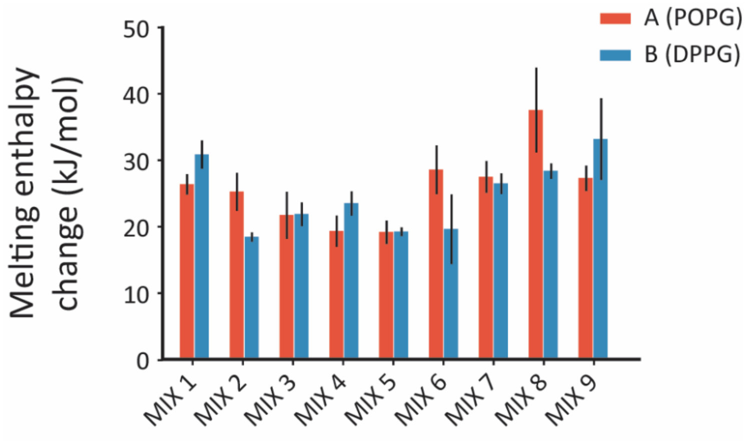

Figure S6: Average melting enthalpy change associated with each DSC main transition peak with standard deviations. The reported values are in line with the expected 20-40 kJ/mol enthalpy change associated with lipid membranes.

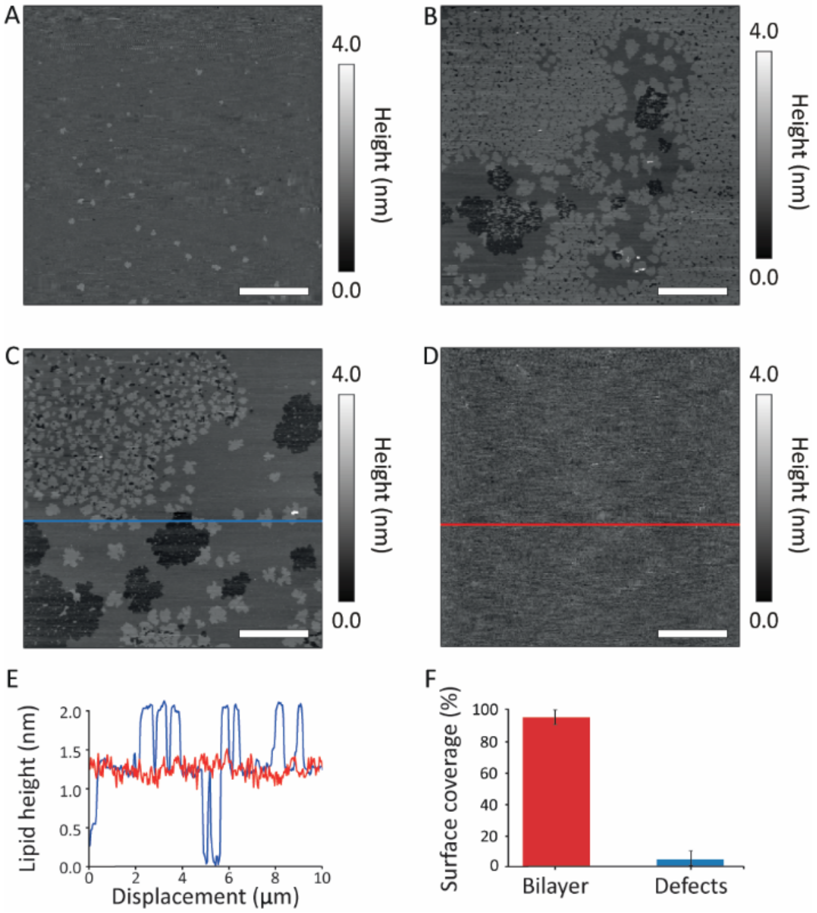

Figure S7: Stability and of SLBs composed of the ternary POPE-POPG-CL mixture at full coverage (0.3 mg/mL SUVs solution for deposition). (A-D) Examples of AFM topographical images of the lipid bilayer before (D – 40°C) and after (A-C – 20°C) the phase transition. (E) Line profiles showing defects in the SLB. The blue profile line is taken from (C) and red from (D). (F) Bar plot representing the average bilayer surface coverage measured over >5 images of SLBs prepared on different days. The scale bare is 7.5 μm in (A), 5 μm in (B) and 2.5 μm in (C-D).

Mixture name	Rupture force F_r (nN)		Young's modulus Y (MPa)	
	**Pre-transition**	**Post-transition**	**Pre-transition**	**Post transition**
**E. coli Native**	3.3 ± 0.3	3.5 ± 0.2	19.0 ± 7.6	24.3 ± 9.0
**E. coli Polar**	2.7 ± 0.3	2.9 ± 0.3	22.0 ± 15.9	40.1 ± 30.3
**MIX – 7A**	3.0 ± 0.2	3.3 ± 0.3	16.9 ± 8.3	22.2 ± 9.9
**MIX – 9A**	2.8 ± 0.3	3.4 ± 0.3	12.2 ± 4.7	16.6 ± 4.7
**POPE**	2.2 ± 0.3	3.1 ± 0.8	55.6 ± 23.3	94.3 ± 39.4
**MIX – 2B**	3.4 ± 0.5	7.1 ± 0.8	28.2 ± 16.8	44.6 ± 14.3

Table S2: Summary of the results obtained from the AFM force maps performed on different lipid mixtures. The average values and associated standard deviation are given for each mixture and each specific lipid phase (liquid ordered and liquid disordered). Images with an even surface coverage of the two phases were selected for the analysis, with a total of 1024 AFM curves per sample. The values for both F_r and Y are the average of approximately 500 indentation curves per lipid phase.

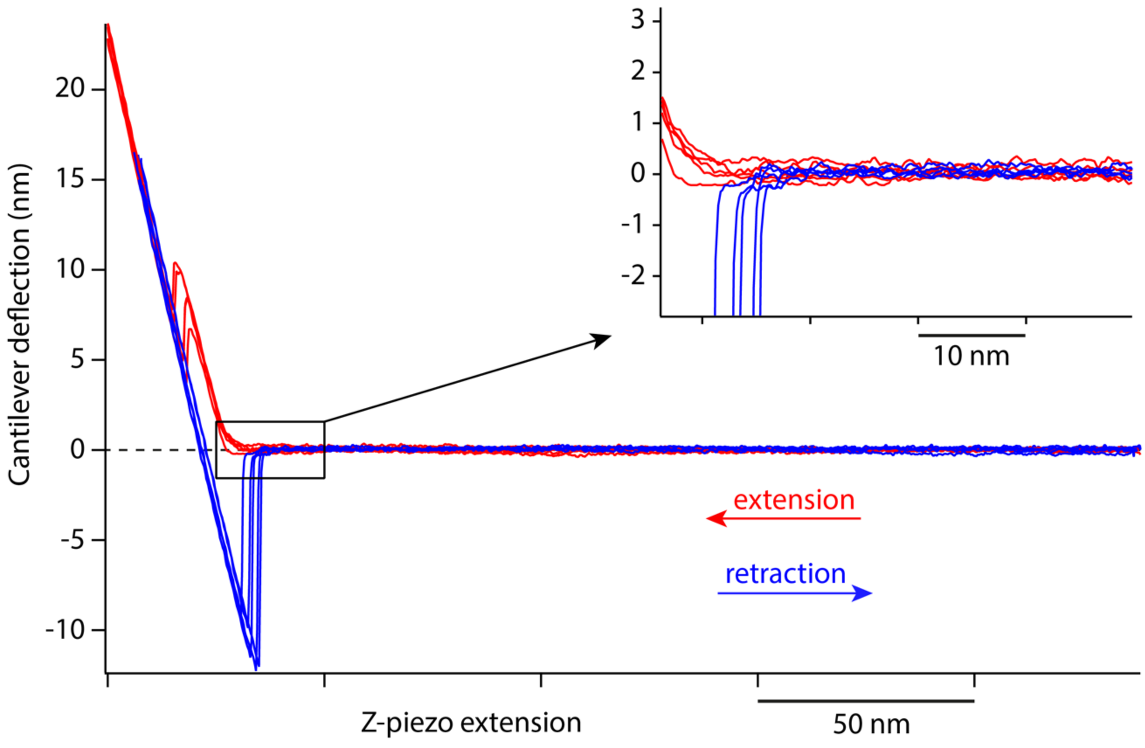

Figure S8: Examples of force curves obtained on different phases and different areas of a SLB, focusing on the adhesion properties of the samples. The approach curves (red) are recorded with the tip moving towards the membrane. The retraction curves (blue) exhibit a strong adhesion to the mica (negative deflection) due to direct tip-mica interactions and the tip getting stuck inside the punctured bilayer. During the initial stages of the retraction, the tip is surrounded by lipids and attached to the bilayer, presumably contributing to the observed adhesion. However, a magnified view of the tip-membrane contact region (inset) shows no clear adhesion beyond that caused by the ruptured membrane. Such adhesion would look like a negative deflection or dip before the tip makes mechanical contact with the sample.

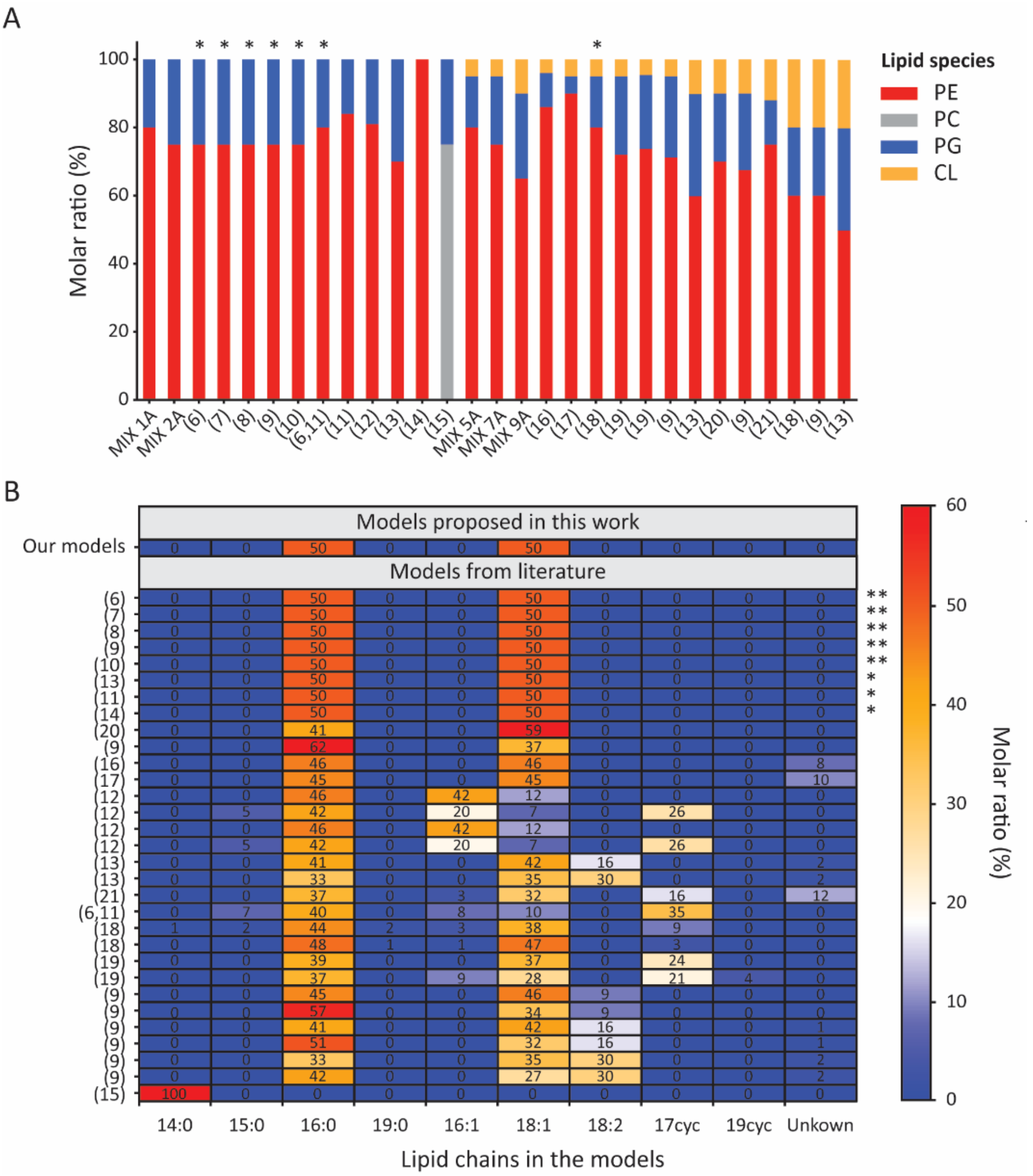

Figure S9: Summary of the different lipid models for *E. coli*/Gram-Negative found in the literature [6-21], and comparison with our ideal binary and ternary model mixtures. Direct comparison across studies is challenging providing the high variability in these models in literature. Moreover, as noted for example by Cetuk et al. [16], part of these models has not been developed to reproduce the properties of native systems, underscoring the risk of drawing conclusions about native membrane behaviour from non-representative models. All the papers have been labelled with their reference number [6-21]. (A) Bar plot showing the lipid headgroup distribution in the pre-existing models. Binary and ternary models have been put side-by-side for clarity. (B) Heatmap representing the acyl chain distributions of the pre-existing models.

* denotes models that match the (A) headgroup distribution or (B) chain distribution of our binary or ternary models.

** denotes models that match both. Interestingly, no ternary model (containing CL) has been identified to match our models' characteristic for both the headgroup and chain distribution.

References

(1) Toda, A.; Hikosaka, M.; Yamada, K. Superheating of the Melting Kinetics in Polymer Crystals: A Possible Nucleation Mechanism. *Polymer (Guildf).* **2002**, *43* (5), 1667–1679. https://doi.org/10.1016/S0032-3861(01)00733-9.

(2) Toda, A. Heating Rate Dependence of Melting Peak Temperature Examined by DSC of Heat Flux Type. *J. Therm. Anal. Calorim.* **2016**, *123* (3), 1795–1808. https://doi.org/10.1007/s10973-015-4603-3.

(3) Garcia, P. D.; Garcia, R. Determination of the Elastic Moduli of a Single Cell Cultured on a Rigid Support by Force Microscopy. *Biophys. J.* **2018**, *114* (12), 2923–2932. https://doi.org/10.1016/j.bpj.2018.05.012.

(4) Hertz, H. Ueber Die Berührung Fester Elastischer Körper. *crll* **1882**, *1882* (92), 156–171. https://doi.org/10.1515/crll.1882.92.156.

(5) Cordina, R. J.; Smith, B.; Tuttle, T. Predicting Lipid Eutectics Using Coarse-Grained Molecular Dynamics. *J. Phys. Chem. B* **2023**. https://doi.org/10.1021/acs.jpcb.3c06297.

(6) Hwang, H.; Paracini, N.; Parks, J. M.; Lakey, J. H.; Gumbart, J. C. Distribution of Mechanical Stress in the Escherichia Coli Cell Envelope. *Biochim. Biophys. Acta - Biomembr.* **2018**, *1860* (12), 2566–2575. https://doi.org/10.1016/j.bbamem.2018.09.020.

(7) Murzyn, K.; Róg, T.; Pasenkiewicz-Gierula, M. Phosphatidylethanolamine-Phosphatidylglycerol Bilayer as a Model of the Inner Bacterial Membrane. *Biophys. J.* **2005**, *88* (2), 1091–1103. https://doi.org/10.1529/biophysj.104.048835.

(8) Seeger, H. M.; Marino, G.; Alessandrini, A.; Facci, P. Effect of Physical Parameters on the Main Phase Transition of Supported Lipid Bilayers. *Biophys. J.* **2009**, *97* (4), 1067–1076. https://doi.org/10.1016/j.bpj.2009.03.068.

(9) Wydro, P. The Influence of Cardiolipin on Phosphatidylglycerol/Phosphatidylethanolamine Monolayers-Studies on Ternary Films Imitating Bacterial Membranes. *Colloids Surfaces B Biointerfaces* **2013**, *106*, 217–223. https://doi.org/10.1016/j.colsurfb.2013.01.053.

(10) Mukherjee, S.; Kar, R. K.; Nanga, R. P. R.; Mroue, K. H.; Ramamoorthy, A.; Bhunia, A. Accelerated Molecular Dynamics Simulation Analysis of MSI-594 in a Lipid Bilayer. *Phys. Chem. Chem. Phys.* **2017**, *19* (29), 19289–19299. https://doi.org/10.1039/c7cp01941f.

(11) Pandit, K. R.; Klauda, J. B. Membrane Models of E. Coli Containing Cyclic Moieties in the Aliphatic Lipid Chain. *Biochim. Biophys. Acta - Biomembr.* **2012**, *1818* (5), 1205–1210. https://doi.org/10.1016/j.bbamem.2012.01.009.

(12) Hsieh, M.-K.; Klauda, J. B. Leaflet Asymmetry Modeling in the Lipid Composition of *Escherichia Coli* Cytoplasmic Membranes. *J. Phys. Chem. B* **2022**, *126* (1), 184–196. https://doi.org/10.1021/acs.jpcb.1c07332.

(13) Luchini, A.; Cavasso, D.; Radulescu, A.; D'Errico, G.; Paduano, L.; Vitiello, G. Structural Organization of Cardiolipin-Containing Vesicles as Models of the Bacterial Cytoplasmic Membrane. *Langmuir* **2021**, *37* (28), 8508–8516. https://doi.org/10.1021/acs.langmuir.1c00981.

(14) Picas, L.; Montero, M. T.; Morros, A.; Oncins, G.; Hernández-Borrell, J. Phase Changes in Supported Planar Bilayers of 1-Palmitoyl-2-Oleoyl- *Sn* -Glycero-3-Phosphoethanolamine. *J. Phys. Chem. B* **2008**, *112* (33), 10181–10187. https://doi.org/10.1021/jp8037522.

(15) Boge, L.; Browning, K. L.; Nordström, R.; Campana, M.; Damgaard, L. S. E.; Seth Caous, J.; Hellsing, M.; Ringstad, L.; Andersson, M. Peptide-Loaded Cubosomes Functioning as an Antimicrobial Unit

against *Escherichia Coli*. *ACS Appl. Mater. Interfaces* **2019**, *11* (24), 21314–21322. https://doi.org/10.1021/acsami.9b01826.

(16) Cetuk, H.; Maramba, J.; Britt, M.; Scott, A. J.; Ernst, R. K.; Mihailescu, M.; Cotten, M. L.; Sukharev, S. Differential Interactions of Piscidins with Phospholipids and Lipopolysaccharides at Membrane Interfaces. *Langmuir* **2020**, *36* (18), 5065–5077. https://doi.org/10.1021/acs.langmuir.0c00017.

(17) Shearer, J.; Marzinek, J. K.; Bond, P. J.; Khalid, S. Molecular Dynamics Simulations of Bacterial Outer Membrane Lipid Extraction: Adequate Sampling? *J. Chem. Phys.* **2020**, *153* (4). https://doi.org/10.1063/5.0017734.

(18) Lopes, S. C.; Neves, C. S.; Eaton, P.; Gameiro, P. Improved Model Systems for Bacterial Membranes from Differing Species: The Importance of Varying Composition in PE/PG/Cardiolipin Ternary Mixtures. *Mol. Membr. Biol.* **2012**, *29* (6), 207–217. https://doi.org/10.3109/09687688.2012.700491.

(19) Pluhackova, K.; Horner, A. Native-like Membrane Models of E. Coli Polar Lipid Extract Shed Light on the Importance of Lipid Composition Complexity. *BMC Biol.* **2021**, *19* (1), 1–22. https://doi.org/10.1186/s12915-020-00936-8.

(20) Dupuy, F. G.; Pagano, I.; Andenoro, K.; Peralta, M. F.; Elhady, Y.; Heinrich, F.; Tristram-Nagle, S. Selective Interaction of Colistin with Lipid Model Membranes. *Biophys. J.* **2018**, *114* (4), 919–928. https://doi.org/10.1016/j.bpj.2017.12.027.

(21) Lind, T. K.; Skoda, M. W. A.; Cárdenas, M. Formation and Characterization of Supported Lipid Bilayers Composed of Phosphatidylethanolamine and Phosphatidylglycerol by Vesicle Fusion, a Simple but Relevant Model for Bacterial Membranes. *ACS Omega* **2019**, *4* (6), 10687–10694. https://doi.org/10.1021/acsomega.9b01075.
